# Bayesian multistate modelling of prefrailty and frailty using microsimulation with weight management interventions for prevention in Asian settings

**DOI:** 10.1038/s43856-026-01756-7

**Published:** 2026-07-04

**Authors:** Xinyu Zhang, Shihui Jin, Haolong Song, Johnathan Hong-Hui Yeo, Borame Lee Dickens

**Affiliations:** Saw Swee Hock School of Public Health, National University of Singapore, and National University Health System, Singapore, Singapore

**Keywords:** Population screening, Lifestyle modification

## Abstract

**Background:**

The prevalence of pre-frailty and frailty is increasing in Asia, resulting in substantial morbidity and mortality in older populations. Modelling frameworks are required to estimate the prevalence of frailty and potential impacts of ongoing population-level nutritional interventions.

**Methods:**

Using a microsimulation sociodemographic model of 3,353,032 individuals from 2011 to 2050, and published estimates from the Singapore Longitudinal Ageing Study 2 of 3270 participants, we developed a Bayesian multistate model of robust, pre-frail, and frail stages with estimated transition probabilities by age, ethnicity, gender, and body mass index category. We then explored four scenarios where weight management interventions are applied that modify the annual distribution of underweight, normal weight, overweight, obese I and obese II individuals.

**Results:**

Here we show that, between 2011 and 2050, the projected overall prevalence of pre-frailty and frailty increases from 44.2% to 49.57% (95% credible interval: 47.60–51.00%), and from 3.2% to 11.97% (7.34–15.60%), respectively. Reductions range from 22,045 (18,430–23,487) to 3,599 (2554–4621) pre-frail individuals and from 62,847 (40,165–91,517) to 15,802 (13,238–17,133) frail individuals when 100% to 5% of both underweight individuals shift to normal weight and obese II individuals move to obese I, respectively. Total healthcare utilisation decreases by 7.1% (3.9–9.4%) to 0.3% (0.2–0.5%) under 100% to 5% intervention coverage.

**Conclusions:**

Frailty prevalence in Singapore is projected to substantially increase by 2050, while population-level weight management interventions could avert cases of both pre-frailty and frailty in older individuals, even at modest real-world effectiveness.

## Plain language summary

As people live longer, more older adults are expected to experience pre-frailty or frailty, which can make everyday activities harder and increase the need for healthcare. Body weight is one factor linked to frailty risk, as both being underweight and having severe obesity can increase this risk. In this study, we used a population simulation model of Singapore residents to project changes in pre-frailty and frailty up to 2050. We also explored whether weight management interventions could reduce this future burden. Our results suggest that frailty is likely to rise substantially in Singapore, but helping older adults move towards healthier weight categories could prevent or delay some cases. These findings support early, population-wide interventions to promote healthy ageing and reduce future healthcare needs.

## Abstract (web summary)

Zhang et al. develop a microsimulation sociodemographic model of 3,353,032 individuals to evaluate the prevalence of frailty in Singapore. Findings show that frailty prevalence in Singapore is projected to substantially increase by 2050 and weight management interventions could reduce this future burden.

## Introduction

Frailty is defined as a clinically recognisable status, resulting from cumulative ageing-associated decline across multiple physiologic systems, which compromises an individual’s ability to perform everyday tasks or cope with acute stressors. As a global public health concern among many countries’ ageing populations, frailty has been associated with increased hospitalisation rates, functional decline, loss of independence, lower quality of life, and mortality^1,2^. The two most widely applied frameworks are the phenotypic model by Fried et al.^3^, which conceptualises physical frailty as a biological syndrome characterised by weakness, exhaustion, unintentional weight loss, slow gait speed, and low physical activity; and the deficit accumulation model by Rockwood and Mitnitski^4^, which describes frailty as the accumulation of health deficits across multiple physiological systems.

A global systematic review of 240 studies between 1998 and 2020 across 62 countries and territories in all United Nations regions, involving 1,755,497 community-dwelling older adults, reported pooled frailty prevalences of 12% (95% Confidence Interval (CI): 11–13%) using physical frailty measures and 24% (22–26%) using deficit-accumulation frailty indices; the pooled prevalences of pre-frailty, a risk status predisposing individuals to frailty, were 46% (45–48%) and 49% (46–52%), respectively, using the same two measures^5^. In Asia, a systematic review of 21 studies between 2015 and 2022, including 52,283 older adults from China, India, Malaysia, Iran, and 10 other Asian countries, reported pooled frailty prevalences of 14.6% (10.9–18.8%) using physical frailty measures and 28.0% (21.3–35.3%) using deficit-accumulation frailty indices^6^. With populations continuing to age, the absolute caseload and public health burden of frailty are expected to rise substantially in the coming decades^7^. Country-specific projections illustrate this increasing burden by showing that in Japan, the frailty prevalence is projected to increase from 11.4% to 14.3% among women and from 7.2% to 8.7% among men aged 60 and above between 2016 and 2043^8^; in Norway, the prevalence of frailty among adults aged 70 and over was 11.7% in 2019 and is projected to nearly double by 2040^9^; and in China, frailty prevalence among older adults increased from 16.9% in 2011 to 22.5% in 2020 and is projected to reach 32.5% by 2030^10^.

Within the Western Pacific, Singapore’s resident population is rapidly ageing^11^. In 2024, the percentage of older persons aged 65 and over accounted for 13.7% of the population, with the number exceeding 0.8 million individuals^12^. By 2050, the proportion is forecasted to reach 23.7%–30.4%, with the number exceeding 1.4 million^13^. The crude prevalence of frailty is therefore expected to rise, although future estimates are scarce. Studies of community-living older adults in Singapore have estimated, however, that the prevalence of pre-frailty and frailty ranged from 40.1% to 44.2% and from 3.2% to 7.5%, respectively, up until 2016^14–16^. The increasing prevalence of frailty poses significant challenges to healthcare systems^17–19^ where those who are underweight or obese, already facing increased likelihoods of complications from injury^20,21^ or chronic diseases^22,23^, are at greater risk^24^.

A meta-analysis of 17 studies including 78,642 community-dwelling older adults from 10 countries in America, Europe, and Asia found that individuals who are obese (BMI ≥ 30 ${kg}/{m}^{2}$) and underweight (BMI < 18.5 ${kg}/{m}^{2}$) were associated with significantly higher risks of frailty (relative risk (RR)  =  1.40, 95% CI: 1.17–1.67 for obesity and RR  =  1.45, 95% CI: 1.10–1.90 for underweight, respectively), and a U-shaped relationship existed between BMI and the risk of frailty with a nadir of risk at a BMI of 18.5–29.9 ${kg}/{m}^{2}\,$^24^. Obesity in particular, which is becoming increasingly prevalent among older adults as sarcopenic obesity^25^, has been linked with frailty in several cross-sectional and prospective population-based studies where a negative relationship has been described^26–30^. The direct mechanisms include increased joint stress and metabolic inflammation, which reduce mobility and lead to the loss of muscle mass and strength. Underweight older adults are also vulnerable via sarcopenia-related mechanisms. Underweight BMI is significantly associated with a higher likelihood of probable sarcopenia (odds ratio (OR) = 2.25, 95% CI: 1.17–4.33)²⁹, and sarcopenia is strongly associated with physical frailty, with pre-frailty and frailty prevalences of 71.7% and 22.6%, respectively, among sarcopenic individuals, compared with 24.7% and 4.0% among non-sarcopenic individuals (*P* = 0.022)^31^. Chronically inadequate nutrition, muscle wasting, and diminished strength in underweight older individuals reinforce the contribution of sarcopenia, a key muscular component, to physical frailty^32^. In terms of interventions, a literature review including 21 studies from the United States, Australia, Canada, and Italy identified strong clinical evidence that weight loss and exercise interventions were effective in improving physical function among obese frail older adults^33^. For underweight older adults, multiple strategies have been taken to prevent weight loss and frailty, and to increase body weight, including supplementing diets with protein, energy, and vitamins in the US^34^ and France^35^.

In Singapore, efforts are underway to decrease frailty rates through multiple weight management and physical health schemes, targeting older adults^36^. A key nutritional intervention is a set of community-based meal delivery schemes, Meals-On-Wheels, aiming to address malnutrition and undernutrition among older adults who may have difficulties in preparing meals at home or accessing local food establishments that may not serve their nutritional needs. The provision of two meals per day, which are nutritionally balanced encouraging healthy eating habits, can support special dietary needs and allow for regular check-ins to monitor the nutritional^37^ status of individuals, and can be utilised for weight management. In such programmes, studies^38,39^ have shown that significant reductions can be observed in overall weight and BMI among those who are overweight, and increases in protein intake and weight gain occurred after meal supplementation among those who are malnourished^40–42^. Through these intervention groups, transitioning underweight individuals to normal weight, and obese individuals to less obese weight categories represent key goals for healthy ageing, but their impact on future pre-frailty and frailty prevalence remains unclear. Such interventions have only been explored in very small-scale studies, which require exploration in large-scale models in order to support decision-making for intervention deployment and potential scale-up.

Our study therefore explored the impact of weight management interventions on projections of pre-frailty and frailty, using Singapore as a case study. First, to estimate the prevalence of pre-frailty and frailty, and explore the impact of interventions on BMI, we built a microsimulation model for 3,353,032 adults aged 55 and above, stratified by gender and ethnicity, including Chinese, Malays, and Indians. Next, we calculated transition probabilities between different frailty statuses using a Bayesian multistate model that accounted for estimated BMI trajectories across each individual’s lifetime. We then modelled three main scenarios examining the change in pre-frailty and frailty prevalence from reducing the proportion of older adults who were underweight, had obesity II, or both.

We show that pre-frailty and frailty prevalence are projected to increase substantially by 2050, but that interventions shifting underweight individuals towards normal weight and individuals with obesity II towards obesity I reduce the projected burden of frailty and associated healthcare utilisation. These findings support early, population-level weight management strategies as part of healthy ageing policy in Singapore and other ageing Asian settings.

## Methods

We built a microsimulation population model to test the effectiveness of interventions designed to reduce frailty among older individuals from 2011 to 2050. We assigned frailty statuses to simulated individuals from 2011 to 2016 using published estimates from two nationally representative studies in Singapore. The first was the Singapore Longitudinal Ageing Study 2 (SLAS-2), which collected detailed data on sociodemographics, anthropometry, physical and cognitive function, chronic diseases, blood biomarkers, and frailty status from a population-based prospective cohort of community-dwelling residents aged ≥ 55 years^14^. The second was the Well-being of the Singapore Elderly (WBSE) study, which collected sociodemographic characteristics and medical conditions from randomly selected Singapore residents aged ≥ 60 years^15^. We then built a Bayesian multistate model to calculate transition probabilities by age, ethnicity, and gender and applied the transition probabilities to individuals across time to obtain nationwide projections of pre-frailty and frailty prevalence from 2011 to 2050. Additionally, we explored the impacts of three weight management interventions on modifying estimated prevalence.

### DEMOS Model

The Demographic Epidemiological Model of Singapore (DEMOS) is an individual-based microsimulation model that generated annual population trajectories for all Singapore residents from 1990 to 2050^43^. In this study, we utilised the population generated by DEMOS, including simulated individuals with assigned frailty statuses in 2011 and 2016, and their sociodemographic characteristics in each year, to estimate frailty transition probabilities and to project nationwide pre-frailty and frailty prevalence from 2011 to 2050 under scenarios with different interventions. The DEMOS leveraged fertility rates, mortality rates, migration, and BMI trajectories from sub-models to project the resident population of Singapore each year (Supplementary Figs. S2 and S3; Supplementary Table S1). We forecasted ethnicity-specific fertility rates using Bayesian Structural Time Series (BSTS) models by MCMC (Markov chain Monte Carlo). We applied a logarithmic transformation to account for the decelerating decline in fertility rates and used annual fertility rates by ethnicity^44^ during periods when fertility rates fell below 2.1 for two consecutive years^45^. Annual age-specific mortality rates from 1990–2023 were obtained from the Singapore Department of Statistics^46^, and mortality rates from 2023–2050 were projected using the Lee-Carter model with logarithmic transformation applied to age-specific mortality rates from 2010–2019. We estimated the yearly migration rates from 1990 to 2020 by comparing simulated and actual populations and projected future migration rates using the mean and variance from 2010–2020. The hierarchical model of BMI trajectories, stratified by gender and ethnicity, described each individual’s BMI over time as Gaussian fluctuations around a sequence of connected lines, with model parameters estimated using statistics from the Singapore Department of Statistics and national surveys^47^.

The population projections were validated by comparing them to the population structure of each one-year age group, gender and ethnicity stratum released by the Singapore Department of Statistics from 1990 to 2023^48^. The projected number of older individuals aged 55 and above in DEMOS was also validated by comparing with the number of Singapore residents published by the Singapore Department of Statistics^48^ from 2011 to 2024 (Supplementary Fig. S1). The projections of BMI were checked by comparing the projected proportions of four BMI categories (underweight (<18.5 ${kg}/{m}^{2}$), normal weight (18.5–24.9 ${kg}/{m}^{2}$), overweight (25.0–29.9 ${kg}/{m}^{2}$), and obese (≥30.0 ${kg}/{m}^{2}$)) with proportions observed in the 2020 and 2022 National Population Health Surveys^49^.

### Identification of frailty status

We adopted Fried’s phenotype criteria, which focus on physical frailty^3^ and assess frailty using five parameters: weakness, slowness, shrinking (unintentional weight loss), exhaustion, and low physical activity. An individual is defined as robust if meeting none of the parameters, as pre-frail if meeting one or two parameters, and as frail if meeting three or more parameters^14^. With synthetic individual data generated by DEMOS from 1990 to 2050, we calculated the total numbers of robust individuals, pre-frail individuals, and frail individuals in 2011 and 2016 based on the prevalence of frailty statuses among Singapore older residents aged 55 years and over from the Singapore Longitudinal Ageing Study 2 (SLAS-2) in 2011 and a 5-year follow-up in 2016^14^, which reported the prevalence of pre-frailty and frailty in 2011 as 44.2% and 3.2%, and as 48.9% and 7.5% in 2016.

Based on the proportions of the robust, pre-frail and frail individuals among older adults across age (55 to 100 years), gender (male, female) and ethnicity (three main ethnicities—Chinese, Malay and Indian), collected from a cross-sectional survey with a nationally representative sample of 2102 Singapore residents using Fried’s phenotype^15^, we estimated the number of individuals in each stratum for robust, pre-frail and frail statuses between 2011 and 2016. We used Bernoulli trials to simulate individual transitions between robust, pre-frail, and frail statuses from 2011 to 2016. Each possible transition was modelled as a binary outcome, in which an individual either made the transition during the period or did not, and the probability of each Bernoulli draw was given by transition probabilities derived from pre-frailty and frailty prevalence in 2011 and 2016.

### Bayesian multistate model

We built a Bayesian multistate model to estimate age-, ethnicity-, and gender-specific annual transition probabilities. We developed our model based on the framework of a Bayesian three-state model, which estimates age-specific incidence and remission rates from cross-sectional population count data using binomial likelihoods and Bayesian smoothing^50^. In our model, frailty progression and remission were assumed to occur only between adjacent states. Within each year, we assumed that a robust individual could stay robust or transition to pre-frailty; a pre-frail individual could revert to robust status, stay pre-frail or transition to frail status; and a frail individual could revert to pre-frail status or stay frail. Direct transitions from robust to frail status or from frail to robust status were not allowed, and at most one transition was assumed to occur within a year. Mortality, however, was not estimated as part of the Bayesian multistate model but was incorporated separately within the DEMOS microsimulation.

We used a two-stage modelling strategy to estimate the transition probabilities. For each gender and ethnicity stratum, in the first stage, we constructed datasets with robust and pre-frail counts to estimate the age-specific five-year transition probabilities from robust to pre-frail status and the remission probabilities from pre-frail to robust status. In the second stage, we constructed datasets with pre-frail and frail counts to estimate the five-year transition probabilities from pre-frail to frail status and the remission probabilities from frail to pre-frail status. The model assumed that the number of individuals making each transition had a binomial likelihood, using the number of individuals at risk as the denominator and the corresponding transition probability as the parameter. We estimated five-year age-, ethnicity-, and gender-specific transition probabilities for older adults at each age from 55 to 85 years, and for age groups 86–87, 88–90, and >90, and then transformed them to annual transition probabilities by the formula

1 $${P}_{1}=1-{\left(1-{P}_{5}\right)}^{1/5}$$

where ${P}_{x}$ represents the transition probability for duration x years.

Transition rates were modelled as smooth functions of age using thin plate regression splines. For each transition, the log transition rate was expressed as a spline function of age, with weakly informative normal priors assigned to the intercept and linear age coefficients, and normal smoothing priors assigned to the nonlinear spline coefficients. The smoothing standard deviations controlled the degree of age-specific variation and were assigned a gamma prior. The corresponding transition probabilities were obtained as deterministic functions of the fitted transition rates within the continuous-time multistate model. The model was implemented using the disbayes R package^51^, which generated posterior samples of transition probabilities using Hamiltonian Monte Carlo sampling. Convergence of model parameters was assessed by examining trace plots and applying the Gelman–Rubin diagnostic to the posterior draws in R. Further mathematical details of the likelihood, spline specification, priors, and model implementation are provided in the Supplementary Information.

### Calibration of estimated prevalence

We applied the annual transition probabilities to individuals aged 55 or over in DEMOS and generated their statuses in the next year using a Bernoulli distribution or categorical distribution based on their assigned statuses in 2011. We repeated such steps and projected the statuses for each individual in the following five years until 2016. As an internal calibration check, we compared the projected prevalence of pre-frailty and frailty in 2016 for older adults aged 60 and over in DEMOS with the observed prevalence for community-dwelling older Singaporeans aged 60 and over in a 5-year follow-up in the SLAS-2^14^.

### BMI intervention modelling

To explore the association between BMI and frailty status in Singapore, we simulated BMI and frailty statuses using summary statistics reported in national surveys in Singapore^14,15^. We classified BMI values into five categories in accordance with the Asia-Pacific classification: underweight (<18.5 ${kg}/{m}^{2}$), normal weight (18.5–22.9 ${kg}/{m}^{2}$), overweight (23.0–24.9 ${kg}/{m}^{2}$), obesity I (25.0–29.9 ${kg}/{m}^{2}$), and obesity II (≥30.0 ${kg}/{m}^{2}$)^52^. We used two logistic regression models to fit the simulated data and calculated the odds ratios of pre-frailty and frailty, respectively, for each BMI category relative to the reference category (normal weight). We found that the majority of individuals were of normal weight (34% of individuals in DEMOS, estimated 33.3% from the SLAS-2 study^14^), so we assumed normal weight to be the reference BMI category. We derived the incidence rate for the normal weight BMI category based on a continuous-time Markov model^53^, such that

2 $${I}_{\left({inc},r\right)}=-\log \left(1-{P}_{\left({inc},r\right)}\right)$$

where ${I}_{\left({inc},r\right)}$ denotes the incidence rate of transitioning to pre-frail or frail status for this BMI category, and ${P}_{\left({inc},r\right)}$ is the corresponding annual incidence probability. Then we applied the original incidence rate between the frailty statuses to the reference BMI category and estimated the incidence rate of any other BMI category i using the formula ${I}_{\left({inc},i\right)}=O{R}_{i}\cdot {I}_{\left({inc},r\right)}$ based on previously established methods^54–56^, which demonstrate that odds ratios can be used to estimate incidence ratios. ${I}_{\left({inc},i\right)}$ denotes the incidence rate of pre-frailty or frailty for the BMI category i, $O{R}_{i}$ denotes the odds ratio for the BMI category i relative to the reference category. The approximate remission probabilities were calculated by similar formulas, but the odds ratio $O{R}_{i}$ was replaced by its inverse. Then we derived the annual incidence probability ${P}_{\left({inc},i\right)}$ for the BMI category i using the previous continuous-time Markov model^53^

3 $${P}_{\left({inc},i\right)}=1-\exp \left(-{I}_{\left({inc},i\right)}\right).$$

### Addition of frailty-related mortality

We utilised a cohort study of 10,912 adults in the Kyoto-Kameoka Study in Japan^57^ to determine a hazard function for mortality based on frailty status and BMI. This study estimated a multivariable-adjusted hazard ratio (HR) between non-frail normal weight (BMI: 21.5–24.9 ${kg}/{m}^{2}$) and other frail status BMI categories, adjusting for age and gender. Thus, we derived the frailty-related mortality rate for each BMI category i using the original mortality rate ${P}_{\left({mortality},r\right)}$ for robust individuals in the reference group and corresponding hazard ratios $H{R}_{i}$ based on the previous continuous-time Markov model^53^

4 $${P}_{\left({mortality},r\right)}=1-\exp \left(-{I}_{\left({mortality},r\right)}\right)$$

We assumed that the hazard ratios of pre-frailty were half of those estimated for frailty based on a systematic review of 24 studies^58^. Utilising ${P}_{\left({mortality},r\right)}$, $H{R}_{i}$, and (4), we estimated the mortality rates for pre-frail and frail individuals in the BMI category i as

5 $${P}_{\left({mortality},i\right)}=1-{({1-P}_{\left({mortality},r\right)})}^{H{R}_{i}},$$

These BMI- and frailty-status-specific annual mortality rates were then applied to each individual to simulate whether they died during that year. Individuals who died were removed from the simulated population and did not contribute to subsequent frailty-status transitions or prevalence estimates. Because mortality primarily affected the number of individuals at risk rather than the transition dynamics between frailty states, its impact on projected frailty prevalence was relatively modest.

### Scenarios

We considered four scenarios based on the established relationship between frailty and BMI: the Baseline Scenario without a weight management intervention where all individuals’ BMI categories remained unaltered as projected in DEMOS in their estimated trajectories; the Underweight Scenario where a proportion (p) of underweight individuals were shifted to normal weight; the Obese II Scenario (Obese II) where p of all obese II individuals were shifted to obese I; the Both Scenario (Both) where p of underweight individuals were shifted to normal weight and p of obese II individuals were shifted to obese I. Here, we set the parameter p to 5%, 10%, 25%, 50%, and 100%, representing differing levels of effectiveness of weight-control interventions, respectively.

### Model output

Based on the assigned frailty statuses in DEMOS in 2011, we projected pre-frailty and frailty prevalence across each stratum under the Baseline Scenario. From 2025 onwards, we adjusted BMI values according to the assumptions of the three scenarios with weight management interventions and projected pre-frailty and frailty numbers and prevalence for each scenario.

### Estimation of healthcare utilisation

To further illustrate the effectiveness of weight management interventions on reducing frailty and associated healthcare costs, we estimated healthcare utilisation in different care settings including specialist outpatient clinic visits (SOCVs), emergency department visits (EDVs), and hospitalisations. We referred to the healthcare utilisation data of community-dwelling older adults aged 60 and over, collected from the Population Health Index study covering two periods: six months before and six months after the observation of frailty statuses^19^. We estimated healthcare utilisation for each year by multiplying the number of robust, pre-frail, and frail individuals by the mean number of SOCVs, EDVs, and hospitalisations expected for each status within a year, which was the sum of the means collected six months before and after the observation, respectively. The six-month means of healthcare utilisation for each status are provided in Supplementary Table S2.

### Statistics and reproducibility

All analyses and visualisations were conducted using R software version 4.4.1^59^. The Bayesian multistate model was implemented using the disbayes package with the RStan backend^51^. The microsimulation included 3,353,032 simulated Singapore residents aged 55 years and above from 2011 to 2050. For each scenario, 12 groups of transition probabilities within their 95% credible intervals (CrIs) were applied to the simulated population in DEMOS, with 30 stochastic projections performed for each group to obtain uncertainty intervals; these stochastic projections were treated as replicates.

### Ethics approval and consent to participate

Human ethics and consent to participate declarations are not applicable to the present modelling study. This study did not involve the collection of new human participant data or the analysis of identifiable individual-level human data. All inputs were derived from previously published aggregated summary statistics, and all analyses were conducted on simulated individuals generated by the DEMOS microsimulation model. The source studies that provided aggregate estimates had obtained the relevant ethics approvals and participant consent. SLAS-2 was approved by the National University of Singapore Institutional Review Board (Ref: 04–140), in accordance with the Declaration of Helsinki and the ethical principles in the Belmont Report, with written informed consent obtained from all participants. The Well-being of the Singapore Elderly study received ethics approval from the National Healthcare Group Domain Specific Review Board and the SingHealth Centralised Institutional Review Board; written informed consent was obtained from participants, or from a legally acceptable representative when participants were unable to provide consent themselves.

## Results

### Transition probabilities

**Fig. 1:**
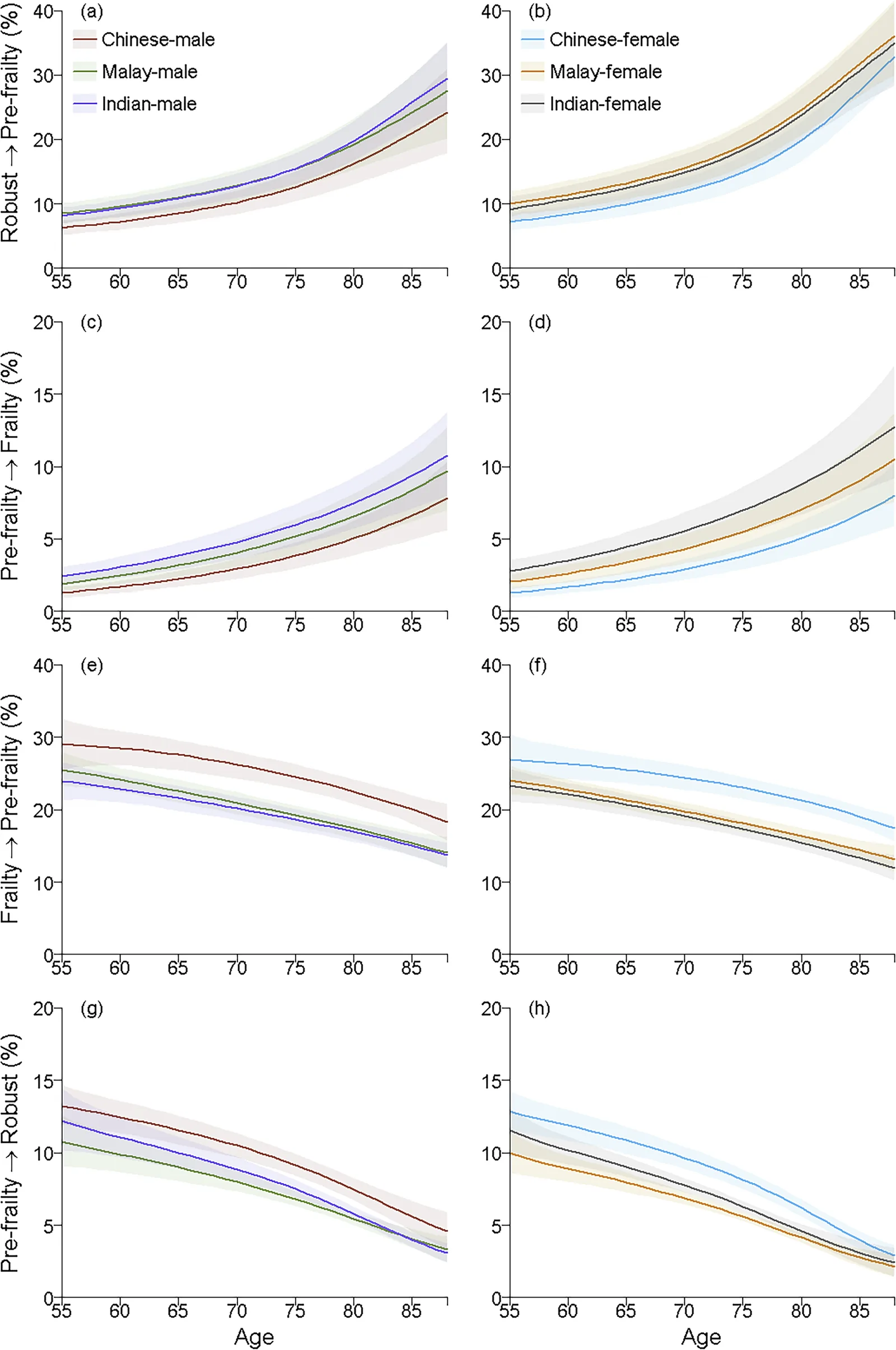
Annual transition probabilities by ethnicity and gender. Posteriors and 95% credible intervals are shown as shaded areas. Panels show transitions from: **a** robust to pre-frail status in men, **b** robust to pre-frail status in women, **c** pre-frail to frail status in men, **d** pre-frail to frail status in women, **e** frail to pre-frail status in men, **f** frail to pre-frail status in women, **g** pre-frail to robust status in men, **h** pre-frail to robust status in women.

Annual incidence and remission probabilities between robust, pre-frailty, and frailty statuses increased and decreased, respectively, with age across all ethnicities and genders (Fig. 1). For Chinese men, the incidence probability of transitioning from robust to pre-frailty rose from 0.063 (95% CrI: 0.052–0.074) at age 55 to 0.242 (0.178–0.309) for those older than 90. Chinese women showed a similar trend, but with higher probabilities, reaching 0.329 (0.290–0.362) for individuals aged over 90. For Malays and Indians, these probabilities were 2.2–4.8% and 1.9–5.3% higher, respectively, than for Chinese. Incidence probabilities between pre-frailty and frailty followed similar age-related trends but were 4.2–27.9% lower and increased more gradually.

Remission probabilities between frailty and pre-frailty decreased with age, from 0.290 (95% CrI: 0.255–0.325) to 0.183 (0.157–0.208) for Chinese men. For Chinese women, these probabilities were lower, declining from 0.269 (0.237–0.304) to 0.174 (0.157–0.192). Malays and Indians had 2.9–5.4% and 3.0–6.1% lower remission probabilities, respectively. Similarly, remission probabilities between pre-frailty and robust declined with age but were 8.8–17.8% lower than those between frailty and pre-frailty.

### Projection results across weight management scenarios

**Fig. 2:**
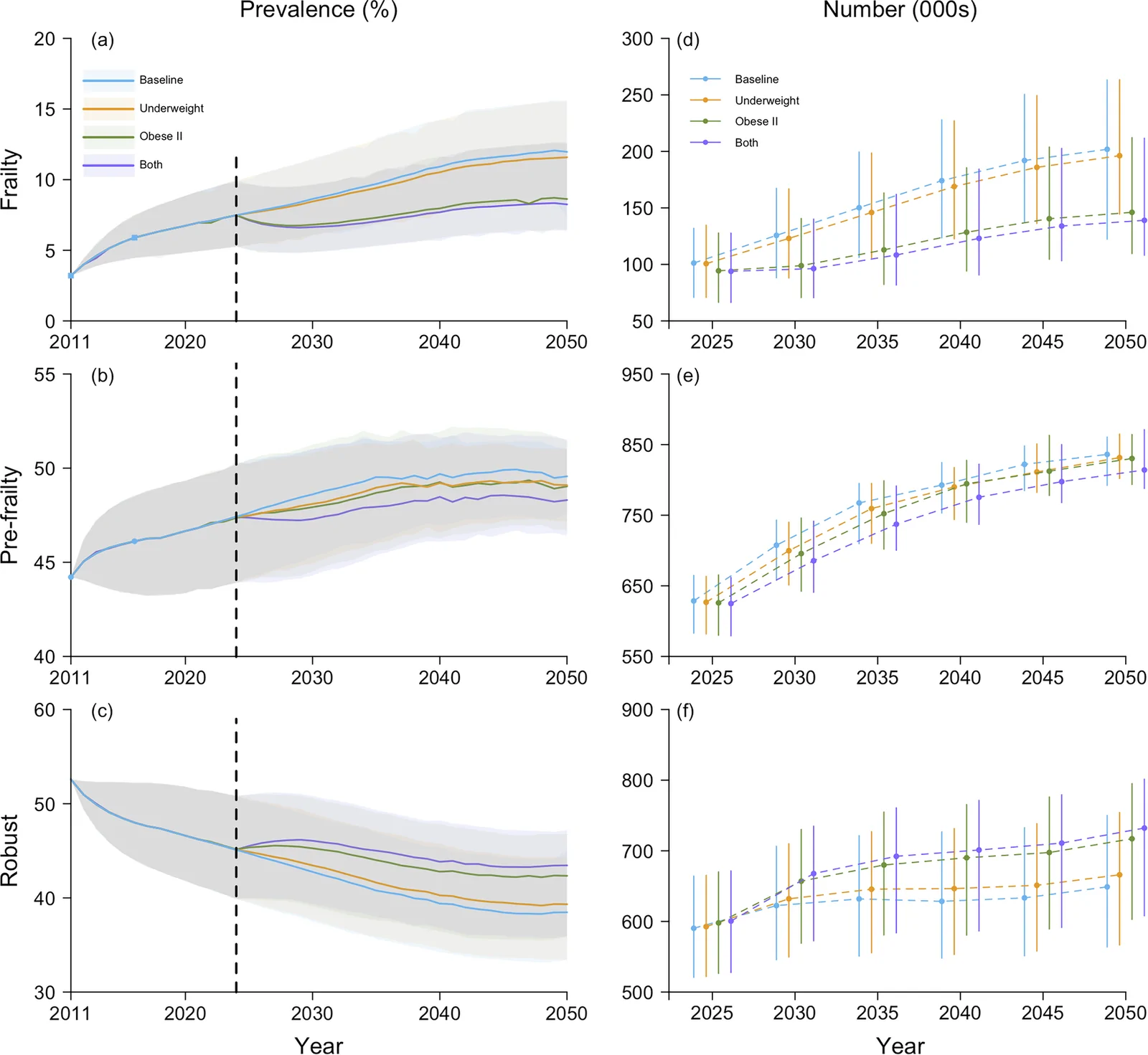
Projected prevalences and population numbers by frailty status under weight management scenarios. Posterior means and 95% credible intervals are shown as shaded areas and error bars for the whole population under four scenarios: baseline, underweight to normal weight intervention (“Underweight”), obese II to obese I intervention (“Obese II”), and the combination of both Underweight and Obese II strategies (“Both”). Panels show: **a** frail prevalence, **b** pre-frail prevalence, **c** robust prevalence, **d** number of frail individuals, **e** number of pre-frail individuals, **f** number of robust individuals. Uncertainty intervals were derived from *n* = 360 stochastic projections per scenario.

In the Baseline Scenario without weight management interventions, the projected pre-frailty prevalence increased from 44.2% to 49.57% (95% CrI: 47.60–51.00%), and frailty prevalence rose from 3.2% to 11.97% (7.34–15.60%) between 2011 and 2050 (Fig. 2). The total numbers of pre-frail and frail individuals grew to 836,117 (792,202–860,643) and 201,859 (122,245–263,199), respectively (Fig. 2), which were 2.3 and 7.8 times higher than those in 2011.

By ethnicity, in 2050, Chinese men had the lowest projected prevalence of pre-frailty (43.8%, 95% CrI: 41.5–44.7%) and frailty (8.1% (5.0–10.7%)), while Chinese women had slightly higher prevalences (49.3% (48.3–49.8%); and 9.5% (5.8%–12.4%)) (Supplementary Fig. S5). For Malays, pre-frailty and frailty prevalences were 1.0–9.1% and 91.5–142.5% higher than those for Chinese. For Indians, pre-frailty prevalence was 5.2% higher for men (0.7–15.4%) but 10.0% lower for women (1.0–15.1%) than for the Chinese ethnicity. Frailty prevalence among Indians generally exceeded that of the Chinese by 128.7–227.3%.

With weight management interventions starting in 2025 at 100% effectiveness, the Both Scenario reduced the number of pre-frail and frail individuals by 22,045 (95% CrI: 18,430–23,487) and 62,847 (40,165–91,517), respectively, by 2050, corresponding to a decrease in pre-frailty and frailty prevalences by 2.6% (0.5–3.2%) and 31.1% (19.6–40.5%) compared to the Baseline Scenario. The Underweight and Obese II Scenarios individually also reduced pre-frail individuals by 4,586 (3305–7313) and 5787 (3670–7707), and frail individuals by 5665 (3205–7,863) and 55,777 (32,683–80,941), respectively, compared to the Baseline Scenario, though these reductions were smaller than those in the Both Scenario.

### Delay in transition of pre-frailty and frailty

**Fig. 3:**
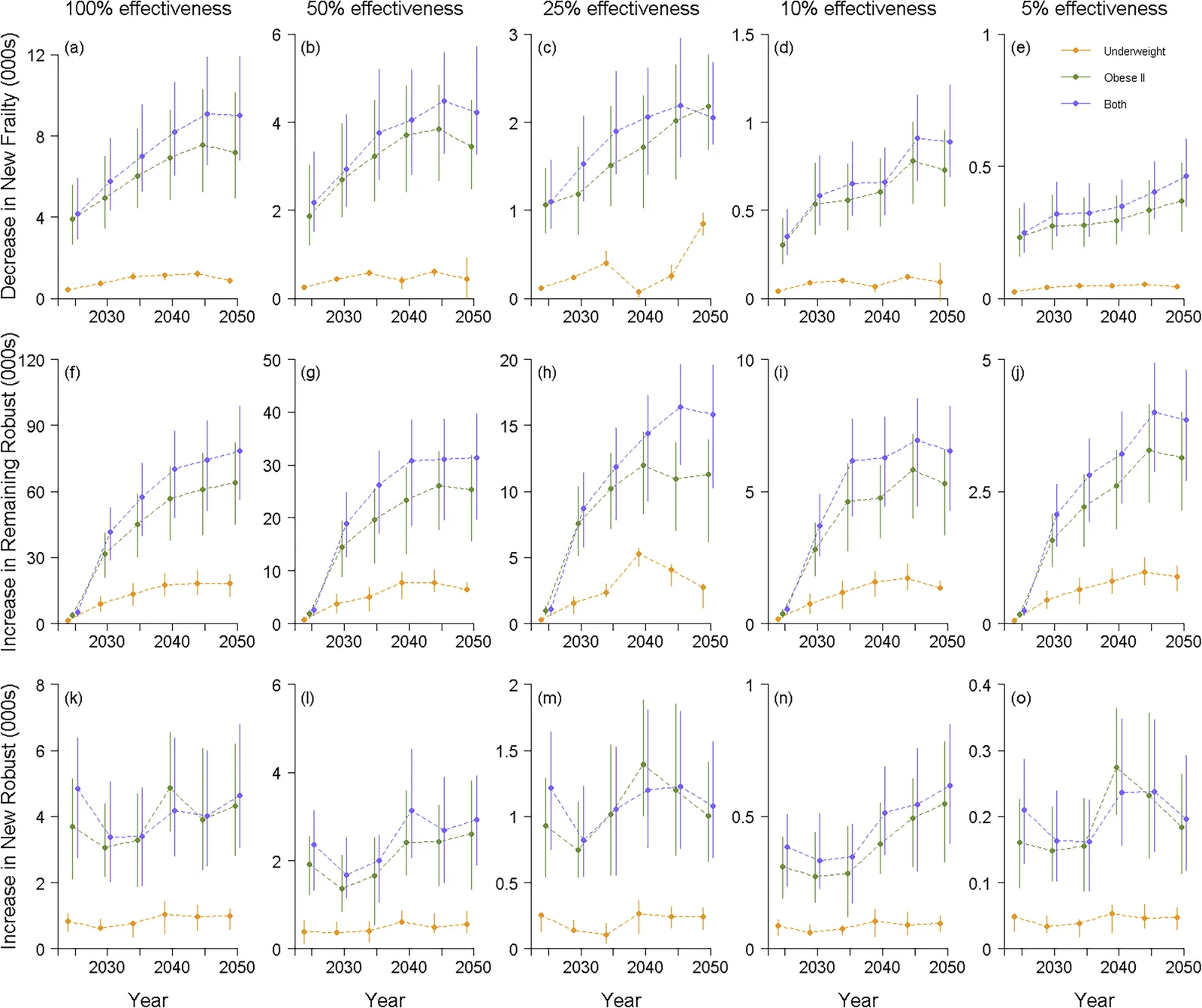
Differences in annual frailty transitions under weight management scenarios. Differences are shown between each scenario with weight management interventions (Underweight, Obese II, Both) and the Baseline scenario from 2025 to 2050. **a**–**e** show changes in the number of new frail individuals under 100%, 50%, 25%, 10%, and 5% intervention effectiveness, respectively. **f**–**j** show changes in the number of remaining robust individuals under 100%, 50%, 25%, 10%, and 5% intervention effectiveness, respectively. **k**–**o** show changes in the number of new robust individuals under 100%, 50%, 25%, 10%, and 5% intervention effectiveness, respectively. Estimates are presented as posterior means and 95% credible intervals shown as error bars. Uncertainty intervals were derived from *n* = 360 stochastic projections per scenario.

**Fig. 4:**
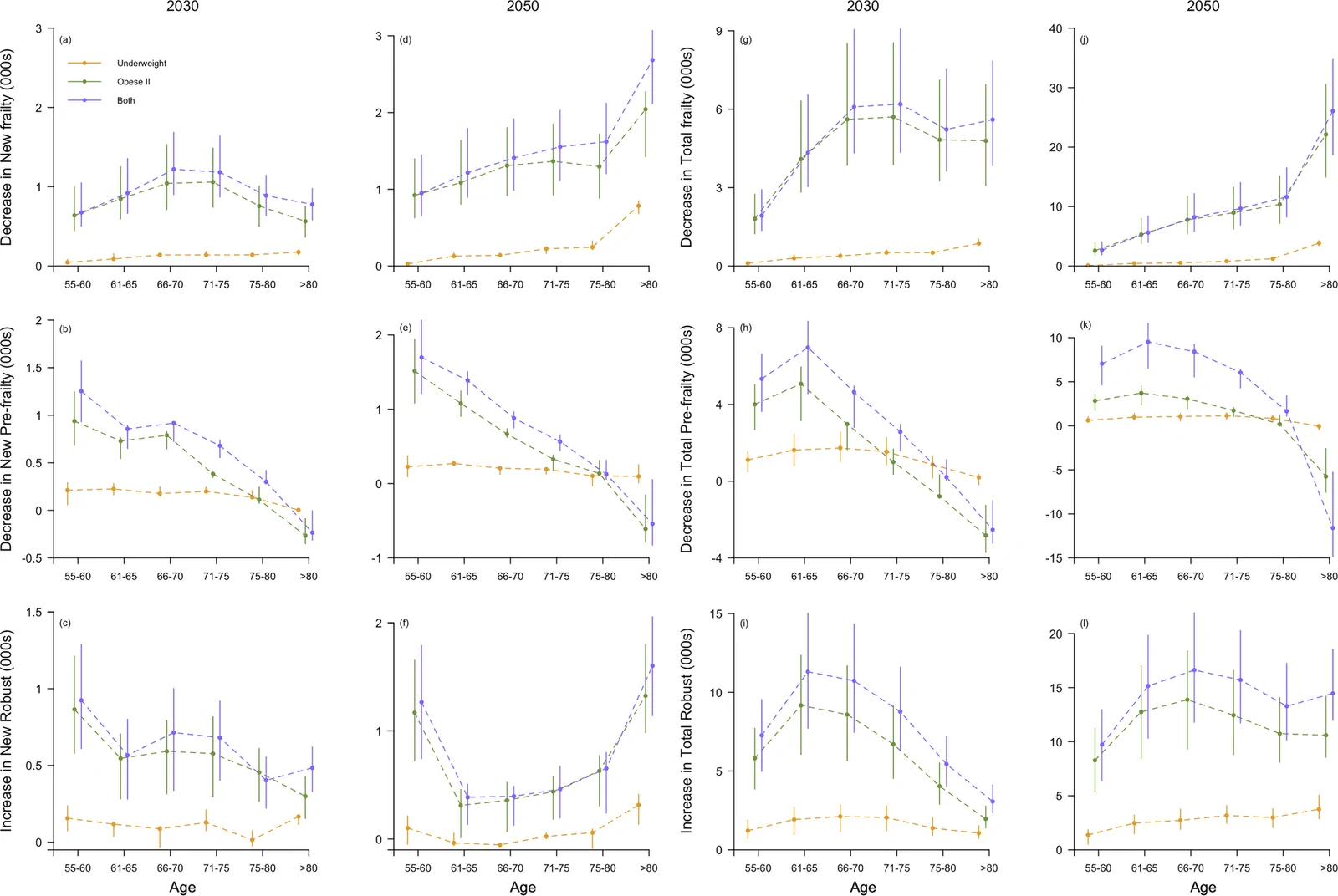
Age-specific changes in frailty status under weight management scenarios. Changes in the number of new and total frail, pre-frail, and robust individuals are shown across age groups under each scenario with weight management interventions (Underweight, Obese II, Both) compared with the Baseline scenario in 2030 and 2050. Estimates are presented as posterior means and 95% credible intervals shown as error bars. Panels show: **a** change in new frail individuals in 2030, **b** new pre-frail individuals in 2030, **c** new robust individuals in 2030, **d** new frail individuals in 2050, **e** new pre-frail individuals in 2050, **f** new robust individuals in 2050, **g** total frail individuals in 2030, **h** total pre-frail individuals in 2030, **i** total robust individuals in 2030, **j** total frail individuals in 2050, **k** total pre-frail individuals in 2050, **l** total robust individuals in 2050. Uncertainty intervals were derived from *n* = 360 stochastic projections per scenario.

We evaluated the effectiveness of BMI interventions by comparing differences in the numbers of new frail individuals, remaining robust individuals, and new robust individuals between scenarios with weight management interventions and the Baseline Scenario from 2025 to 2050 (Fig. 3), and the changes in new and total frail, pre-frail, and robust individuals by age group composition in 2030 and 2050 (Fig. 4). Five alternative scenarios were considered in Fig. 3, where interventions were effective for 100%, 50%, 25%, 10%, and 5% of target individuals.

From 2025 to 2050, the Baseline Scenario showed a yearly increase in new frail individuals, rising from 24,558 (95% CrI: 17,809–31,472) to 41,019 (31,127–51,542). All scenarios with interventions reduced the number of new frail individuals, with reductions increasing over time, even in the case of 5% effectiveness. The Both Scenario achieved the largest reduction, with 9,013 (6796–11,895), 4228 (3282–5714), 2052 (1749–2680), 891 (692–1210), and 464 (350–603) fewer new frail individuals by 2050 under intervention effectiveness levels of 100%, 50%, 25%, 10%, and 5%, respectively.

Similarly, the Both Scenario yielded the highest number of remaining robust individuals, exceeding the Baseline Scenario (544,390 (95% CrI: 463,578–643,190) remaining robust individuals) by 78,263 (56,444–98,788), 31,407 (19,834–39,673), 15,793 (10,235–19,562), 6542 (4260–8220), and 3,848 (2711–4805) by 2050 under scenarios with 100%, 50%, 25%, 10%, and 5% intervention effectiveness, respectively. For individuals reverting to robust status, all scenarios showed increases over time across the five intervention effectiveness levels. The Both Scenario achieved the largest increase, exceeding the Baseline Scenario (66,936 (64,647–69,899) new robust individuals) by 4637 (3065–6784), 2933 (1899–3931), 1082 (690–1565), 618 (397–849), and 197 (118–292), respectively.

In 2030 and 2050, all intervention scenarios mitigated frailty by reducing new and total frail individuals while increasing new and total robust individuals across all age groups compared to the Baseline Scenario (Fig. 4). The reductions in both new frail individuals and total frail individuals showed increasing trends with age in 2050. Among individuals older than 80 years, the Both Scenario achieved the highest reductions of 33.8% (95% CrI: 30.7–37.1%) and 25.3% (24.9–26.5%), with 2685 (2116–3068) and 26,064 (18,696–34,876) fewer new frail and total frail individuals compared to the Baseline scenario (7905 (6063–9654) new frail individuals and 102,803 (75,669–131,031) total frail individuals).

### Healthcare utilisation analysis

**Fig. 5:**
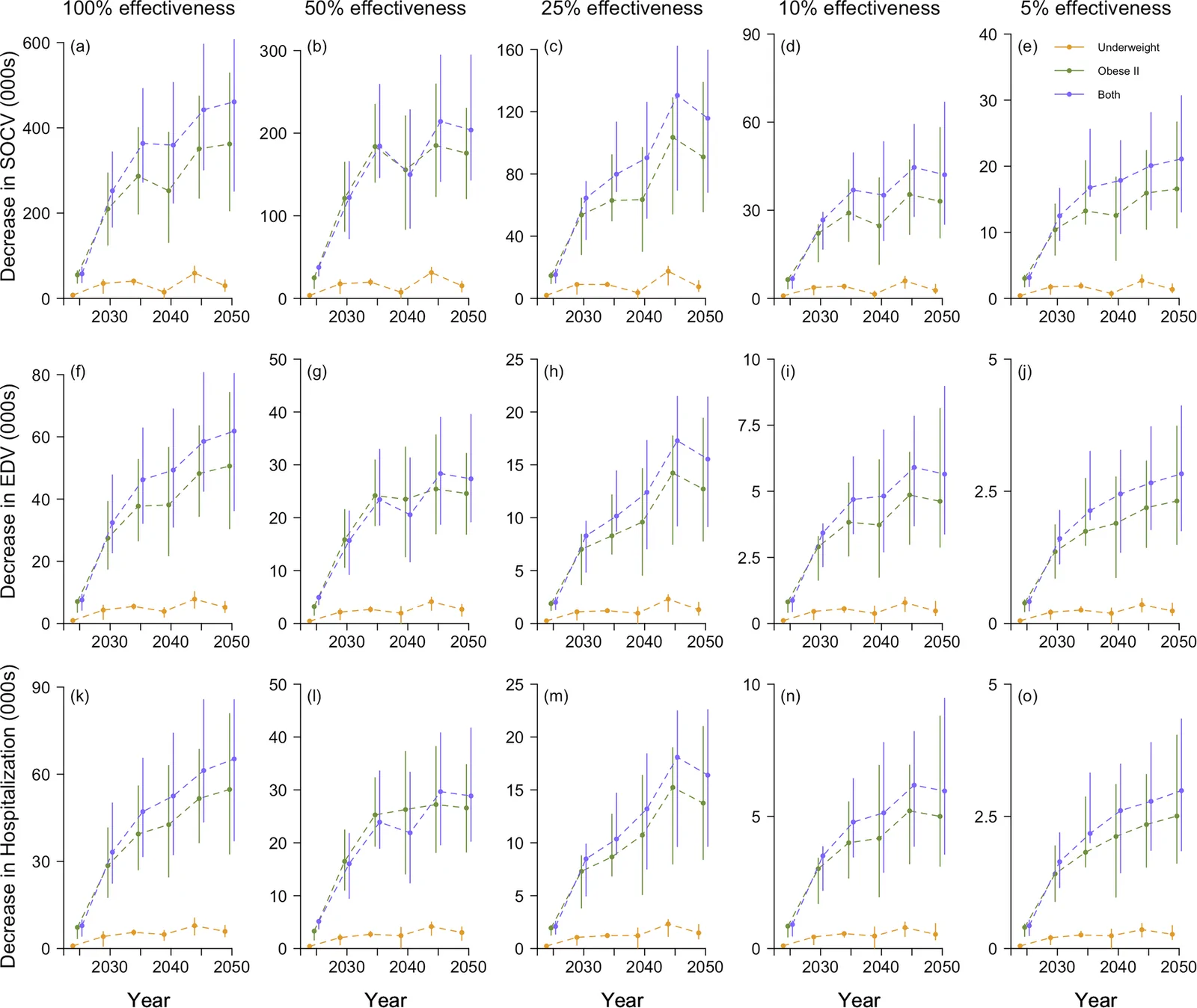
Reductions in healthcare utilisation under weight management scenarios. Reductions are shown for SOCVs, EDVs, and hospitalisations in each scenario with weight management interventions (Underweight, Obese II, Both) compared with the Baseline scenario from 2025 to 2050. **a**–**e** show reductions in SOCVs under 100%, 50%, 25%, 10%, and 5% intervention effectiveness, respectively. **f**–**j** show reductions in EDVs under 100%, 50%, 25%, 10%, and 5% intervention effectiveness, respectively. **k**–**o** show reductions in hospitalisations under 100%, 50%, 25%, 10%, and 5% intervention effectiveness, respectively. Estimates are presented as posterior means and 95% credible intervals shown as error bars. SOCV, specialist outpatient clinic visits; EDV, emergency department visits. Uncertainty intervals were derived from *n* = 360 stochastic projections per scenario.

The Both Scenario achieved the largest reduction (Fig. 5), with 460,914 (95% CrI: 251,312–607,011), 203,836 (143,225–294,561), 115,801 (68,189–159,570), 42,088 (25,211–66,840), and 21,097 (13,064–30,670) fewer SOCVs under 100%, 50%, 25%, 10% and 5% weight management intervention effectiveness scenarios, respectively, compared to the Baseline Scenario, where the number of SOCVs was projected to be 7,097,602 (5,979,538–8,003,813) by 2050. Similarly, the number of EDVs showed rising annual decreases. By 2050, the Both Scenario reached the highest reduction with 61,858 (36,295–80,364), 27,356 (19,222–39,532), 15,541 (9152–21,416), 5649 (3384–8971), and 2831 (1753–4,116) fewer EDVs across the five intervention effectiveness levels, respectively, compared to the Baseline Scenario, which projected 610,145 (499,172–697,157) EDVs by 2050. Hospitalisations followed the same trends, with larger annual decreases across all intervention scenarios. By 2050, the Both Scenario generated the largest reduction with 65,263 (37,069–85,674), 28,862 (20,280–41,708), 16,397 (9655–22,594), 5959 (3570–9464), and 2,987 (1,850–4,343) fewer hospitalisations across the five different intervention effectiveness scenarios compared to the Baseline Scenario, which projected 472,748 hospitalisations (372,569–549,991).

## Discussion

In this study, we constructed a microsimulation model to forecast the prevalence of pre-frailty and frailty in an ageing population of 3,353,032 people and found significant increases in both by 2030 and 2050. We examined the impact of weight management interventions that are widely utilised as primary and secondary measures to avert these outcomes. We discussed the effectiveness of these strategies in mitigating frailty risks and highlighted the implications for public health policies aimed at reducing frailty prevalence and enhancing healthcare outcomes.

We estimated that 707,535 (95% CrI: 657,925–743,032) and 125,686 (88,168–167,322) of all individuals across age, ethnicities and genders will be pre-frail and frail by 2030, increasing up to 836,117 (792,202–860,643) and 201,859 (122,245–263,199) by 2050 respectively, corresponding to 2.3- and 7.8- fold increases in population size compared with 2011. Outside of Southeast Asia, a systematic review of various longitudinal studies from 1987 to 2020 examining frailty trajectories in the USA, China, the Netherlands, Ireland, the UK, Sweden, and other European countries found that frailty prevalence has been increasing^60^. A modelling study in Australia estimated that in 2016, approximately 415,769 people aged 65 years or more were frail and almost 1.7 million people were pre-frail, reaching 609,306 frail and 2,248,977 pre-frail individuals by 2027^61^. In England, simulation projections indicated that frailty will increase by 7.1%, from 41.5% to 48.7% between 2017 and 2027^62^.

Our transition estimates also revealed notable ethnic and gender differences. Chinese older adults, particularly men, were less likely to transition to more severe frailty statuses, consistent with different frailty prevalences for three ethnicities shown in the WBSE study^15^. Healthier dietary patterns, greater dietary diversity, higher levels of physical activity, and lower prevalence of high-risk BMI reported in Chinese cohorts help to explain these differences^63–65^. The lower transition probabilities for men align with evidence that women experience higher frailty risk^15,66^, potentially due to lower muscle mass and strength, and post-menopausal changes affecting bone and muscle health^67–69^. Transition probabilities into pre-frailty were higher in contrast to transitions into frail and robust statuses, aligning with longitudinal evidence that pre-frailty is a dynamic and reversible status, whereas frailty is more severe and less likely to fully reverse once established^70^.

Reducing frailty prevalence is a public health priority as frailty results in adverse health outcomes, increased hospitalisation rates and an overall decline in the quality of life with higher healthcare utilisation. Frailty is potentially reversible; therefore, identifying and addressing frailty earlier can benefit both individuals and healthcare systems^71^. A randomised controlled trial (RCT) in Singapore involving 149 older adults found that six-month nutritional supplementation and cognitive training interventions significantly reduced frailty scores compared to the control group, with odds ratios (OR) of 2.98 and 2.89, respectively, and decreased frailty prevalence by 16–22%; beneficial effects were observed at three and six months and persisted at 12 months, demonstrating that targeted interventions can mitigate frailty with sustained long-term effectiveness in older adults^72^. Within a three-month intervention study and four-month follow-up in Japan among 131 frail older women, those assigned to the milk fat globule membrane supplementation and exercise group had a frailty reversal rate of 45.5%^73^. An RCT in Spain implemented a six-month interdisciplinary intervention involving Mediterranean diet advice among 200 pre-frail older adults between 2016 and 2017. After 12 months, the intervention group showed significant frailty mitigation (RR 2.90; 95% CI 1.45–8.69) and higher reversion to robust status (14.1% vs. 1.1%, *p* < 0.001)^74^.

We found that the prevalence of frailty among underweight and obese II increased from 5.3% and 4.8% in 2011, to 11.2% (95% CrI: 7.9–14.7%) and 23.1% (17.6–28.1%) by 2050, respectively. Similar associations between BMI values and frailty prevalence have been observed in other studies. In a cross-sectional study in Japan with 7,191 participants in 2011-2012, the ORs for frailty prevalence were 2.04 (1.58–2.63) for the underweight and 1.54 (1.15–2.07) for the obese^75^. Additionally, a study with 4019 participants from the Netherlands between 2008 and 2012 found a statistically significant U-shaped association between BMI and physical frailty^76^ after adjusting for sex, age, level of education, and smoking status, with the prevalence of physical frailty being 8.2% in underweight participants, 2.9% in normal weight participants, 2.6% in overweight participants, and 5.0% in obese participants. Furthermore, meta-analyses involving over 23 thousand individuals from Chile, Spain, England, America, Finland, and Australia (spanning 1974–2022) found that both obese (OR = 1.74, *p* < 0.01) and underweight (OR = 1.70, *p* < 0.01) older adults had a higher risk of frailty than those with normal weight using random effects models^77^.

In our study, with weight management for both the underweight and obese II groups, we estimated a reduction in pre-frailty and frailty by 22,045 (95% CrI: 18,430–23,487; reduction: 2.6% (0.5–3.2%)) and 62,847 (40,165–91,517; reduction: 31.1% (19.6–40.5%)) by 2050. In the USA, an RCT of 27 obese frail volunteers in 2003 found that a six-month calorie-restricted diet aimed at creating an energy deficit of about 750 kcal/day led to 8.4% ± 5.6% body weight loss and significant improvements in frailty scores assessed by the Physical Performance Test score (2.6 ± 2.5 vs 0.1 ± 1.0; *P* < 0.05), peak oxygen consumption (1.7 ± 1.6 vs 0.3 ± 1.1 mL/min per kilogram; *P* < 0.05), and Functional Status Questionnaire score (2.9 ± 3.7 vs −0.2 ± 3.9; *P* < 0.05) in the treatment group^78^. Between 2012–2014, the MEASUR-UP RCT in the USA implemented the intervention of a six-month reduced-calorie diet at two protein levels among 67 obese II individuals, improving physical composite and physical functioning scores by 9.2 and 12.7 (both *p* < 0.001) at six months^79^. In France, an RCT across retirement homes found that dietary supplements increased muscle power by 57% at three months (*p* < 0.05) with an average BMI increase of 3.65%^80^. Relatively modest weight changes, such as gaining or losing less than five kilograms, correspond to the substantial majority of transitions from underweight to normal weight and from obese II to obese I, which are achievable for most individuals through long-term, community and clinical weight management programmes in Singapore^81–83^. Under the purview of Healthier SG, which has enrolled over one million adults aged above 40 into its enhanced screening and GP-patient tracking programme^84^, sustained BMI changes are feasible under a long-term national implementation scheme.

Our study supports the need for early identification^85^ and weight management interventions among older individuals to reduce frailty progression rates. An RCT conducted in Taiwan from 2015 to 2017 for 319 pre-frail and frail older adults showed that at the six-month measurement, the frailty scores significantly improved in both the nutrition group (difference from the baseline: −0.28; 95% CI: −0.41,−0.11) and the combined exercise and nutrition group (−0.34; 95% CI: −0.52,−0.16) compared to the control group^86^. In a study of 738 adults aged 60 and above in Singapore, 90.5% exceeded sugar intake, 68.5% of men and 57.1% of women exceeded saturated fat intake, and 49.5% and 55.3% had inadequate dietary calcium intake, respectively. The study also found that 22.3% of these older adults were at moderate to high malnutrition risk^87^. Programmes such as the Healthier Dining Programme (HDP)^88^, which increases access to healthier food and drink options by offering grants to food and beverage businesses to promote healthier meal options, supporting many older populations that regularly dine at food stalls, could be further developed to provide more fresh produce, less saturated fat, and more protein within typical meals. Counselling and diet management programmes with general practitioners (GPs) under Healthier SG^89^, an initiative that aims for GPs to follow individuals through their lifetime for long-term physical health monitoring, could also be utilised to encourage weight management among older adults. Among the underweight, additional oral supplementation through subsidised schemes could be utilised, as a study of 811 participants aged 65 or over who underwent this intervention had an average of 5% weight gain and increased physical strength^90^. Policy recommendations could additionally focus on personalised nutrition programmes with targeted subsidies for nutrient-dense meal plans, particularly benefiting underweight seniors, at food stalls as well as within Meals on Wheels to target a wider population^91^. Community-based weight management centres could be established to improve access to structured interventions such as dietary education, cooking workshops, and peer-led support programmes. Routine BMI and frailty screenings would also be highly recommended to enable early detection and timely intervention at the robust and at-risk of becoming pre-frail stage. An integrated plan targeting malnutrition among older adults within the community, workplace, and at home is urgently needed, with necessary healthcare support to maintain robustness and keep individuals at a healthy BMI, which has been shown to be an important factor in preventing and averting frailty.

A further critical point is that frailty management cannot begin only once individuals become frail, and effective prevention must start earlier in the life course. In the context of BMI management, implementing prevention efforts among adults while they are still robust, particularly those who are underweight or in obese II categories, can meaningfully reduce their risks of transitioning to pre-frailty and frailty. Although ageing will inevitably lead many older adults to develop some degree of vulnerability, lowering risk earlier and delaying progression can improve population health, quality of life, and reduce healthcare burdens. Evidence from Singapore supports the feasibility of sustained, population-level BMI improvement under structured programmes. For underweight older adults, the Strengthening Health In Elderly through nutrition (SHIELD) RCT involving 811 individuals aged ≥65 demonstrated that six months of nutritional supplementation and counselling led to ≥5% weight gain for a large proportion of participants with concurrent functional improvements^92^. For overweight and obese adults, community programmes delivered by the Health Promotion Board, such as Lose To Win, achieved average 1.3–3.6 kg weight loss over 12 weeks, with follow-up support enabling many participants to maintain healthier weight trajectories^93^. Singapore’s Obesity Clinical Practice Guidelines similarly summarise international evidence showing that comprehensive lifestyle and medical interventions can sustain up to 10% weight loss over 1–5 years^94^. This underscores the need for scalable, population-level programmes incorporated into routine care in Singapore, similar in structure to Healthier SG, rather than relying on small intervention trials or recruitment into specialised frailty programmes. Embedding BMI checks, brief counselling, and appropriate nutritional or weight management interventions within standard clinic visits would allow sustained engagement across the population and provide a practical pathway for shifting BMI trajectories and mitigating frailty progression at scale.

Our research has several limitations. We assumed that the proportions of robust, pre-frail and frail adults and the remission between each status from the Singapore Longitudinal Ageing Study 2 (SLAS-2) in 2011 and a 5-year follow-up in 2016^14^ were representative of the whole population, with a five-year follow-up period. We also utilised age, gender, ethnicity, and BMI as the main factors in determining frailty risk, as we lacked data on the potential additional risk of other covariates such as smoking, physical activity, dietary habits, chronic diseases, education levels, and socioeconomic factors. In this context, BMI may partly reflect broader underlying health conditions, such as chronic diseases, nutritional status, and muscle function, that are not explicitly included in the model. As such, the estimated BMI-specific effects should be interpreted as capturing overall BMI-related risk rather than a purely causal effect of BMI alone. In addition, BMI interventions were implemented as shifts between BMI categories without explicitly modelling individual-level weight trajectories or regain. This can be interpreted as reflecting repeated intervention with varying long-term effectiveness, where effectiveness levels represent the proportion of individuals who sustain healthier BMI categories over time. Our estimates of healthcare utilisation did not account for heterogeneity in the impact of frailty across individuals and were based on frailty-specific mean utilisation values from community-dwelling older adults in Singapore applied across the simulated population over time. As utilisation patterns may differ across socioeconomic groups over time, the projected reductions serve as an overall population-level estimate with the intervention and treatment being applied uniformly across the population based on current treatment methods and costs. Although frailty captures overall health status and is closely related to multimorbidity, future work could incorporate disease-specific pathways and socioeconomic stratification, where data permit, to further refine healthcare utilisation estimates. Although we utilised a data-driven approach, we simplified the model to estimate prevalence of pre-frailty and frailty. Although excess BMI, including extreme obesity, has been associated with increased frailty risk in multiple studies^24^, several additional indirect pathways also exist, such as the interaction between diabetes and frailty, which requires further exploration. The bidirectional causality of frailty and comorbidities also merits investigation in additional studies. In addition, our population projections may not truly reflect future trends as policy changes may alter migration and fertility rates. However, our model’s frailty prevalence estimates aligned with findings from a large study conducted in 2021 by the Singapore Ministry of Health Data Analytics Division that collected and analysed data from 425,293 seniors aged ≥60 years (estimated: 6.9%, our study: 8.2% [5.8–11.1%])^95^. Data on frailty and pre-frailty estimates are scarce not only in Singapore but in the wider region of the Western Pacific, making modelling studies imperative for evidence-based decision-making, although such analyses should be repeated as new prevalence estimates become available over time to improve the accuracy of projections and estimates of intervention effectiveness. As this study was based on Singapore, a high-income country, the quantitative estimates may not be directly generalisable to settings with different dietary patterns, nutritional conditions, healthcare access, and baseline frailty risks. However, the overall findings are applicable as the need for urgent intervention with globally increasing pre-frailty and frailty prevalences is clear, which is further compounded by increasingly poor nutrition availability for seniors in recent decades. The modelling framework could also be applied to other countries using appropriate local demographic and frailty risk profiles for tailored estimates and strategies. Finally, because the SLAS-2-based frailty transition estimates are likely most applicable to community-dwelling older adults, institutionalised older adults and those with very severe frailty or dementia may have been under-represented in the analytic sample. As institutionalised older adults may differ from community-dwelling older adults in baseline frailty, care environment, and support needs, future work should incorporate data from institutional long-term care settings to assess whether frailty prevalence and transition parameters differ in these populations and to extend the model accordingly.

This study developed a microsimulation model using available public data, estimated transition probabilities accounting for key risk factors, and assessed the impact of weight management interventions, which are widely implemented globally. The prevalence of pre-frailty and frailty was projected to substantially increase by 2030 and 2050, while population-level weight management interventions could help avert cases of both pre-frailty and frailty in older individuals. Our results provide policymakers with fitted national-level projections of elderly pre-frailty and frailty in scenarios with weight management interventions, which enable the quantification of uncertainties in pre-frail and frail burdens. Our findings also support the development of health policies and services that help older adults shift from underweight or obese II BMI categories towards normal weight or obesity I, respectively, thereby reducing frailty risks in Asian populations. It is also worth highlighting that our model is sufficiently generic to be applied to other ageing settings, given the availability of relevant demographic statistics and frailty prevalence estimates elsewhere.

## Supplementary information

Supplementary Information

Description of Additional Supplementary files

Supplementary Data 1

Supplementary Data 2

Supplementary Data 3

Supplementary Data 4

Supplementary Data 5

## Data Availability

The publicly available data used in this study can be accessed through the links to public domain resources provided in the article and Supplementary Information. Source data underlying Figs. 1–5 in the manuscript are provided as Supplementary Data Figs. 1–5. Additional aggregated data generated during the current study are stored on secure institutional servers and are available from the corresponding author on reasonable request. Requests should be directed to the corresponding author, Borame Lee Dickens, at ephdbsl@nus.edu.sg, and will be reviewed in accordance with the data-sharing policies of the corresponding author’s institution. The corresponding author will respond to data requests within two weeks of receipt.
