# Supplementary material for: Bayesian multistate modelling of prefrailty and frailty using microsimulation with weight management interventions for prevention in Asian settings
Source: Commun Med (Lond). 2026 Jul 4;6:560. doi: 10.1038/s43856-026-01756-7 (PMC13713980; doi:10.1038/s43856-026-01756-7)
Supplement: Supplementary file 1 [file 43856_2026_1756_MOESM1_ESM.pdf]

Supplementary Information

Sample Size

The total sample size of 3,353,032 in our microsimulation model covered individuals aged 55 and above across three main ethnicities—Chinese, Malays and Indians in Singapore from 2011 to 2050. We utilised the Demographic Epidemiological Model of Singapore (DEMOS) to generate individual data, representing each resident in Singapore (including citizens and permanent residents) from 1990 to 2050. Next, we projected the frailty statuses for each older individual aged 55 and above in DEMOS from 2011 to 2050.

We validated the sample size by comparing the total number of older individuals aged 55 and above in DEMOS with the number of Singapore residents published by the Singapore Department of Statistics[1] from 2011 to 2024 in Fig. S1. The two numbers aligned, indicating that DEMOS' modelled estimates reflect the population counts for Singapore.

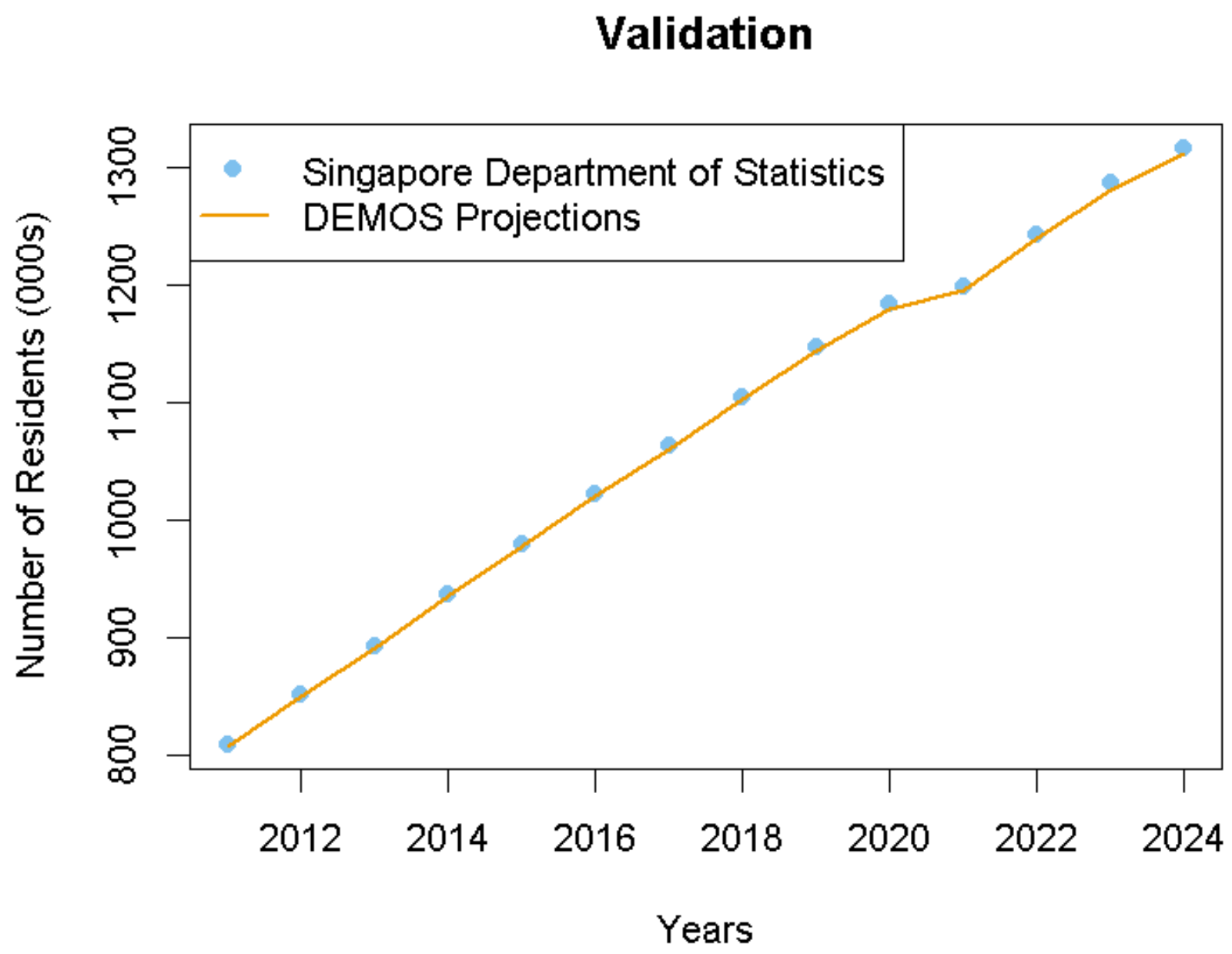

Figure S1. The number of older residents aged 55 and above in Singapore: comparison between Singapore Department of Statistics data and DEMOS data from 2011 to 2024.

Migration Assumptions

Regarding migration, we assumed a migration rate with small variance from 2012 onwards. Using published fertility rates and mortality rates[2,3], we estimated the yearly migration rates from 1990 to 2020 in Fig. S2. For future projections, we calculated the mean and variance of migration rates from the 2010–2020 period and simulated future migration by applying the mean with variance we calculated previously.

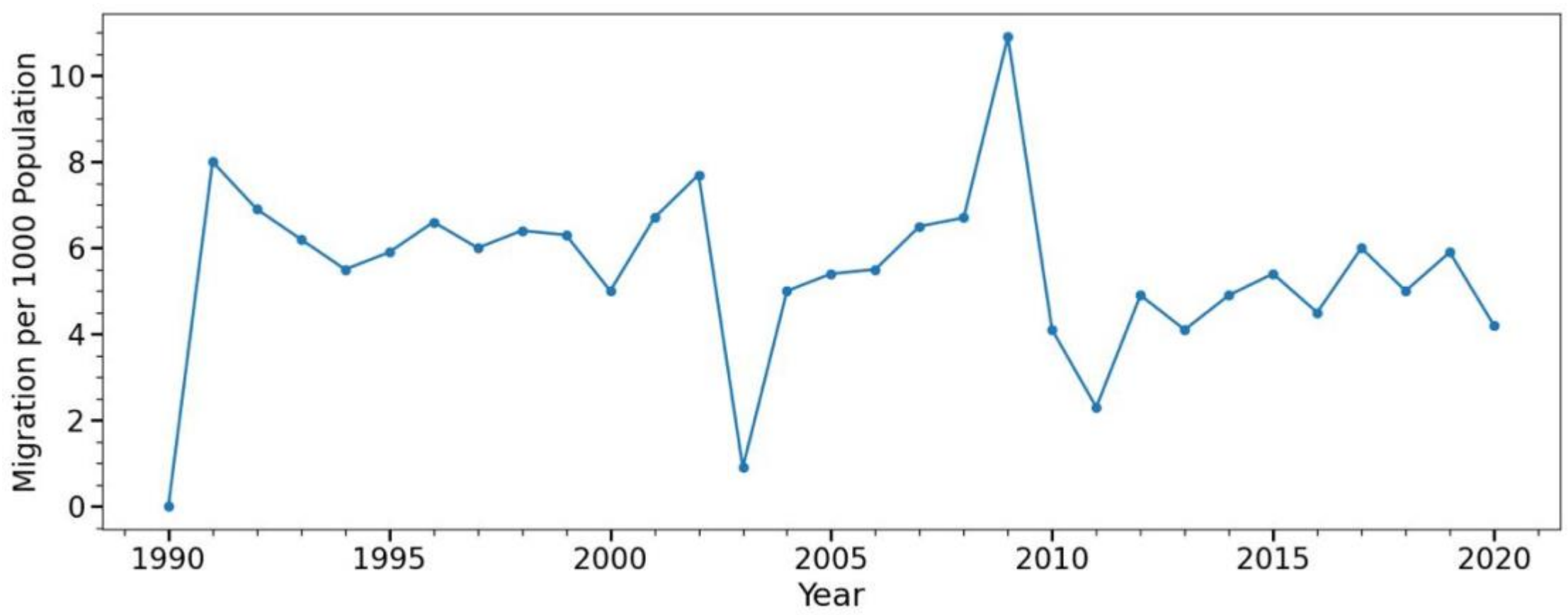

Figure S2. Singapore migration trends (number of migrants per 1,000 residents) for 1990-2020.

Fertility Rates Model

We forecasted ethnicity-specific total fertility rates using a Bayesian structural time-series (BSTS) framework[4]. Separate models were fitted for the three major ethnic groups in Singapore, Chinese, Malay, and Indian residents, because historical fertility patterns differed across these three major ethnic groups in Singapore. For residents outside these groups, fertility rates were approximated using the Malay fertility series, as the residual ethnic group was small and showed similar observed fertility values. Model fitting used annual ethnicity-specific fertility rates from the low-fertility phase. This phase was defined as beginning in the year when two consecutive declines in fertility led the rate to fall below the replacement-level threshold of 2.1. It covered 1975–2022 for Chinese and Indian residents and 2003–2022 for Malay residents. Fertility rates were analysed on the logarithmic scale to capture the slowing rate of decline during this phase.

For each ethnic group, let Y_t denote the logarithm of the total fertility rate in year t. In the BSTS model, the observation equation was written as

$$Y_t = \mu_t + c_t + \epsilon_t, \epsilon_t \sim N(0, \sigma_\epsilon^2) \quad (1)$$

where μ_t is the latent trend component, c_t is an optional cyclic component, and ϵ_t is the observation error. The cyclic component c_t was included only for Chinese fertility rates, where the historical series exhibited an approximately 12-year periodic pattern. For Malay and Indian fertility rates, c_t was set to zero.

For Chinese and Indian residents, the latent trend was modelled using a semi-local linear trend model. In this model, the level component follows a random walk, while the slope component follows an autoregressive process centred on a long-run slope:

$$\mu_{t+1} = \mu_t + \delta_t + \eta_{\mu t},\ \eta_{\mu t} \sim N\left(0, \sigma_\mu^2\right), \quad (2)$$

$$\delta_{t+1} = D + \phi(\delta_t - D) + \eta_{\delta t}, \eta_{\delta t} \sim N\left(0, \sigma_\delta^2\right), \quad (3)$$

Where δ_t is the local slope in year t, D is the long-run slope, ϕ controls the persistence of deviations from the long-run slope, and $\eta_{\mu t}$ and $\eta_{\delta t}$ are zero-mean Gaussian state innovations. The standard deviations σ_μ and σ_δ control the variability of the level and slope components, respectively.

For Malay residents, a simpler AR(1) state component was used because the observed time series was shorter and showed a relatively simpler temporal pattern. We describe this as an AR(1) state component rather than specifying a separate explicit trend equation, as it was used only to provide a parsimonious trend representation for the Malay fertility series.

Prior distributions were specified for the state components following the BSTS semi-local linear trend framework. Inverse gamma priors were assigned to the level standard deviation σ_μ and the slope standard deviation σ_δ. A Gaussian prior was assigned to the long-run slope parameter D, and a potentially truncated Gaussian prior was assigned to the AR(1) coefficient ϕ, with truncation to the stationary interval when stationarity of the slope deviations was imposed.

For each ethnicity, we split the time series into training and validation periods, and 5,000 posterior draws were generated using Markov Chain Monte Carlo (MCMC) sampling. The first 10% of iterations were discarded as burn-in to reduce the influence of initial values. Posterior predictive means were used as point estimates. Model performance was assessed using mean absolute percentage error in the training and validation periods, demonstrating high prediction accuracy across the testing sets (Table S1). Convergence for the BSTS fertility models was assessed by examining posterior trace plots and by conducting the Heidelberger–Welch convergence diagnostic tests on the posterior draws in R.

Table S1. Model performance, measured by mean absolute percentage error (MAPE) for the three BSTS models.

	Training set (year)	Validation set (year)	$\text{MAPE}_{\text{Train}}$ (%)	$\text{MAPE}_{\text{Valid}}$ (%)
Chinese	1975–2010	2011–2022	4.76	3.71
Malays	2003–2017	2018–2022	5.91	4.69
Indians	1975–2010	2011–2022	3.33	4.77

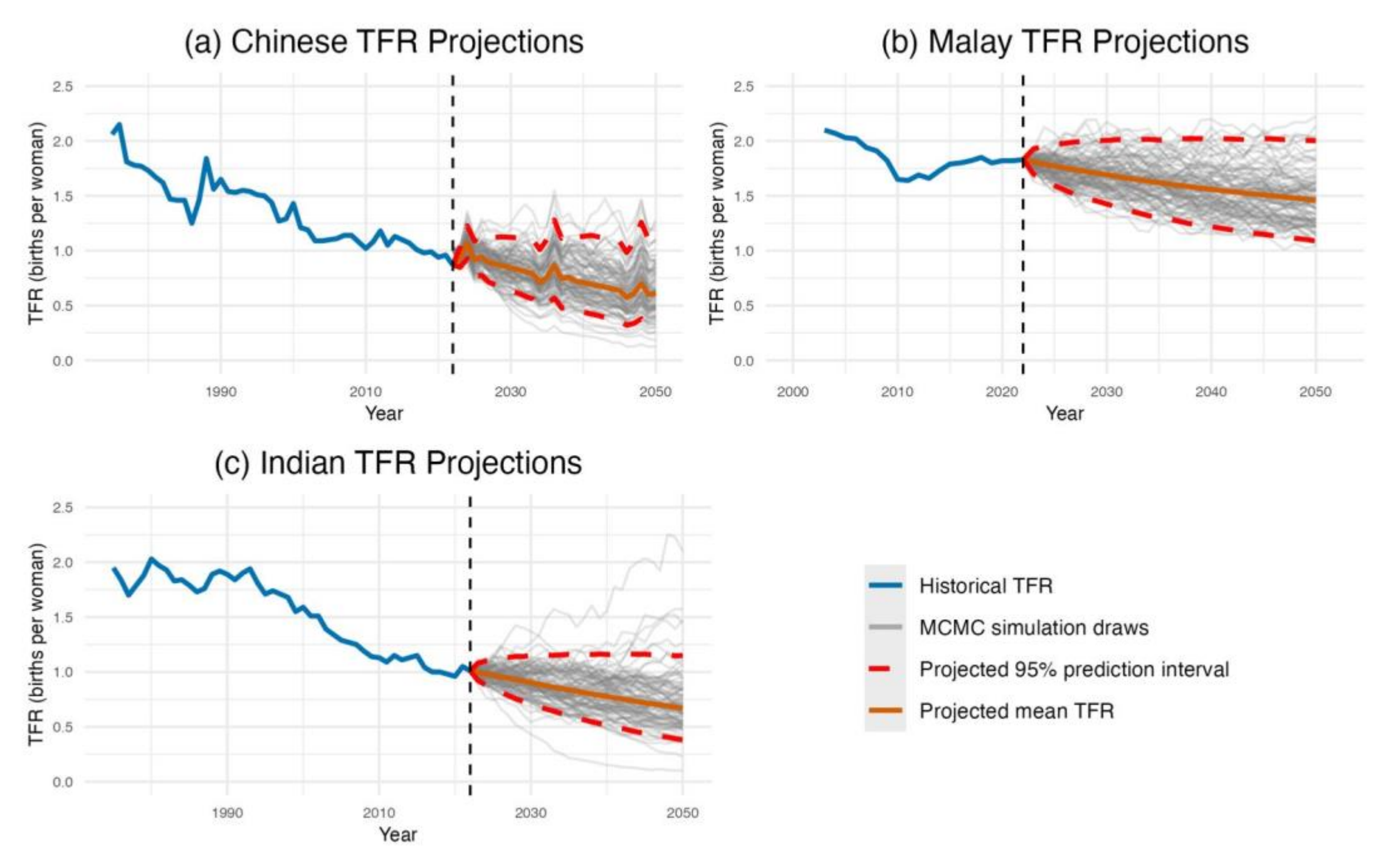

Figure S3. Projected total fertility rates for Chinese (a), Malays (b), and Indians (c) from 2023 to 2050, with grey lines representing 100 MCMC simulation draws.

Bayesian Multistate Model

The original multistate model proposed by Jackson et al. consists of three states, health, disease, and death, and assumes transitions from health to disease, from disease to death, and from disease back to health, with the schematic structure [Health] ↔ [Disease] → [Death][5]. This model is implemented in the disbayes R package[6], which uses observed cross-sectional data on disease counts from finite populations to conduct Bayesian inference for age-specific incidence rates, remission rates, case fatality rates, prevalence, and other related parameters. Based on this framework, we developed a Bayesian multistate model to estimate age-specific transition probabilities between robust, pre-frail, and frail states using the disbayes package in R, stratified by gender and ethnicity.

Our approach focuses on the progression and remission within adjacent frailty states and excluded mortality or case fatality from the analysis. We adopted a two-stage modelling strategy with the schematic structure [Robust state] ↔ [Pre-frail state] ↔ [Frail state] in Fig. S4, using two separate datasets to estimate transition probabilities between robust and pre-frail states, and between pre-frail and frail states.

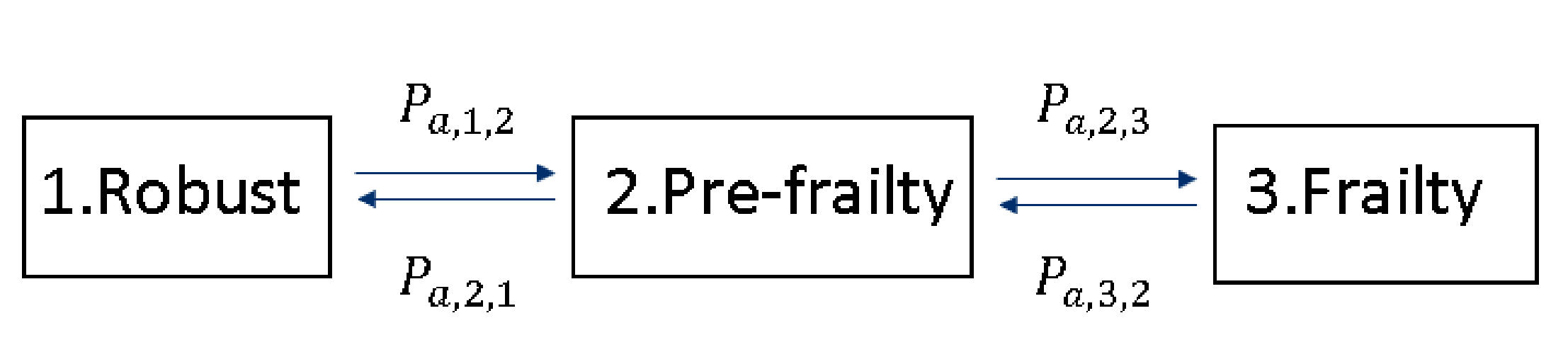

Figure S4. Bayesian multistate model with robust, pre-frail, and frail states and transition probabilities from robust to pre-frail state ($P_{a,1,2}$), from pre-frail to robust state ($P_{a,2,1}$), from pre-frail to frail state ($P_{a,2,3}$), and from frail to pre-frail state ($P_{a,3,2}$) for individuals at each age a.

The model assumes that each individual may transition only between adjacent states and at most once per year. In the first stage, for each gender and ethnicity stratum, we constructed a dataset including the number of individuals at risk (i.e., robust individuals) ($n_a^{(inc,1)}$) at each age a, the number of incident cases of pre-frailty within the next five years ($y_a^{(inc,1)}$), the number of pre-frail individuals ($n_a^{(rem,1)}$) at each age a, and the number of individuals reverting from pre-frail to robust state between 2011 and 2016 ($y_a^{(rem,1)}$). The dataset was used in the disbayes package to estimate the five-year transition probabilities from robust to pre-frail state and from pre-frail to robust state. In the second stage, we constructed a dataset focused on pre-frail and frail counts, including the number of pre-frail individuals at each age a ($n_a^{(inc,2)}$), the number of incident cases of frailty ($y_a^{(inc,2)}$), the number of frail individuals $n_a^{(rem,2)}$, and the number of remissions from frail to pre-frail state between 2011 and 2016 ($y_a^{(rem,2)}$). The second dataset was used to estimate the five-year transition probabilities from pre-frail to frail state, and from frail to pre-frail state.

The model assumes that observed counts have binomial likelihoods, separately for the incidence data $y_a^{(inc,i)}$ and $n_a^{(inc,i)}$ and the remission data $y_a^{(rem,i)}$ and $n_a^{(rem,i)}$, for $i = 1, 2$:

$y_a^{(inc,1)} \sim Binomial\left(n_a^{(inc,1)}, P_{a,1,2}\right)$, where $P_{a,1,2}$ is the five-year incidence probability from robust state to pre-frail state.

$y_a^{(rem,1)} \sim Binomial\left(n_a^{(rem,1)}, P_{a,2,1}\right)$, where $P_{a,2,1}$ is the remission probability from pre-frail state to robust state.

$y_a^{(inc,2)} \sim Binomial\left(n_a^{(inc,2)}, P_{a,2,3}\right)$, where $P_{a,2,3}$ is the five-year incidence probability from pre-frail state to frail state.

$y_a^{(rem,2)} \sim Binomial\left(n_a^{(rem,2)}, P_{a,3,2}\right)$, where $P_{a,3,2}$ is the remission probability from frail state to pre-frail state.

These binomial likelihoods were specified separately for the two adjacent-state submodels. The first-stage robust–pre-frail submodel included the progression counts from robust to pre-frail status and the remission counts from pre-frail to robust status. The second-stage pre-frail–frail submodel included the progression counts from pre-frail to frail status and the remission counts from frail to pre-frail status. The two adjacent-state models were fitted separately, and their estimated transition probabilities were then combined for use in the DEMOS simulation.

Each log transition rate is modelled as a smooth function of age using thin plate regression splines[7]:

$$\log(q_{a,i,j}) = \sum_{k=1}^{K} \beta_{i,j,k} g_{i,j,k}(a), \text{or compactly: } \log(q_{i,j}) = G_{i,j} \cdot \beta_{i,j}, \quad (5)$$

for transitions $(i,j) = (1,2),(2,1),(2,3),(3,2)$, where $q_{a,i,j}$ denotes the age-specific transition rate from state i to state j, $g_{i,j,k}(a)$ are spline basis functions, including the intercept term $g_{i,j,1}(a) = 1$, the slope term $g_{i,j,2}(a) = a$, and the remaining terms $g_{i,j,k}(a)$ for $k \geq 3$ which capture the nonlinear relationship; $\beta_{i,j,k}$ are coefficients to be estimated, with a common prior distribution $\beta_{i,j,k} \sim N(0, \lambda_{i,j,0}^2)\ for\ k \geq 3$. The basis matrix $G_{i,j} \in R^{A \times K}$ is precomputed, with each row corresponding to an age a and each column representing the evaluation of basis function $g_{i,j,k}(a)$ for $k = 1, \ldots, K$ across all ages. Thin plate regression splines are specified following Wood's formulation[7], involving radial basis functions and low-rank approximations derived from thin plate spline theory. They are constructed by applying eigen decomposition to the penalty matrix associated with the spline's roughness penalty functional, which selects the dominant smooth components of the function space[8]. The corresponding transition probabilities, denoted by $P_{a,i,j}$, which were used in the binomial likelihood, were obtained as deterministic functions of the fitted transition rates within the continuous-time multistate model.

Unlike traditional splines, thin plate splines do not use explicitly defined knots and manual knot selection is unnecessary. We use K=10 basis functions, which provided sufficient flexibility for modelling smooth age patterns across the age range considered, and the prior distribution $N(0, \lambda_{i,j,0}^2)$ on $\beta_{i,j,k}\ for\ k \geq 3$ serves as a Bayesian smoothing penalty by shrinking unnecessary terms towards 0, preventing overfitting. The prior standard deviation $\lambda_{i,j,0}$ controls the degree of smoothness, with smaller $\lambda_{i,j,0} \rightarrow 0$ increasing linearity of the curve and large $\lambda_{i,j,0}$ allowing more flexibility. Bayesian updating of $\lambda_{i,j,0}$ enables the model to adapt smoothness to the data. The prior Gamma(2,1) assigned to $\lambda_{i,j,0}$ favours moderate values of $\lambda_{i,j,0}$, resulting in moderate smoothness and preventing extreme shrinkage. For the intercept $\beta_{i,j,1}$ and slope $\beta_{i,j,2}$, weakly informative normal priors $N(0, 100^2)$ are assigned to allow the model sufficient flexibility to fit a wide range of plausible baseline levels and trends while avoiding overly extreme values in the absence of data support.

The model is implemented via the disbayes package, which uses the RStan backend to perform Bayesian inference through Hamiltonian Monte Carlo (HMC). Posterior samples of the spline coefficients $\beta_{i,j,k}$ from which the age-specific transition rates and corresponding probabilities are derived, are generated through the following steps:

1. Precompute the spline basis matrix $G_{i,j}$, and initialize all parameters, including spline

coefficients $\beta_{i,j}^{(0)}$, and smoothness hyperparameters $\lambda_{i,j,0}^{(0)}$ for each transition $(i,j) = (1,2), (2,1), (2,3), (3,2)$.

2. For each MCMC iteration $t = 1, \ldots, T-1$:

   (1) Compute the log transition rates using the current spline coefficients: $log(q_{i,j}^{(t)}) = G_{i,j} \cdot \beta_{i,j}^{(t)}$, convert them to positive rates $q_{i,j}^{(t)} = \exp(G_{i,j} \cdot \beta_{i,j}^{(t)})$, and map these rates to transition probabilities $P_{i,j}^{(t)}$ within the continuous-time multistate model.

   (2) Compute the log posterior using the likelihood of the observed data and the priors on spline coefficients $\beta_{i,j,k}$ and hyperparameters $\lambda_{i,j,0}$, and update parameters of interest using the gradient of the log posterior distribution.

These computations are handled internally by the disbayes package during MCMC sampling, which generates samples from the posterior distribution for parameters including the transition probabilities from the robust state to pre-frailty ($P_{a,1,2}$), from pre-frailty to robust state ($P_{a,2,1}$), from pre-frailty to frailty ($P_{a,2,3}$), and from frailty to pre-frailty ($P_{a,3,2}$). Convergence of the Bayesian multistate model parameters is assessed by examining trace plots of posterior draws and applying the Gelman–Rubin diagnostic to the posterior draws in R.

As individual transition histories between 2011 and 2016 were not directly observed, transition probabilities were estimated using five-year changes in frailty status between the two SLAS-2 waves. Therefore, estimation required several structural assumptions. First, transitions were restricted to adjacent frailty states, so direct movement from robust to frail status or from frail to robust status within a single step was not allowed. Second, at most one transition was allowed within each year. In addition, the model was fitted in two stages, separately for robust ↔ pre-frail and pre-frail ↔ frail transitions, which reduced the number of transition parameters estimated simultaneously. Finally, age-specific transition probabilities were not estimated independently at each age, but were modelled as smooth functions of age using thin plate regression splines, which borrowed strength across neighbouring ages and regularised estimation. Together, these assumptions constrained the parameter space and supported estimation of transition probabilities from the two observation timepoints. We further assessed the reliability of the estimated transitions by comparing projected pre-frailty and frailty prevalence in 2016, and frailty prevalence in 2021, with the corresponding observed prevalences in Singapore.

Risk Factors in the Pre-frailty/Frailty Model

We used a sample of 1,051 community-dwelling older adults in a cohort study, which showed that the prevalence of pre-frailty and frailty significantly increased with age (P<0.001), and differed by gender (P=0.01) and ethnicity (P=0.048)[9], providing supporting evidence on frailty-associated

factors. BMI was also significant (P<0.001) in the SLAS-2 sample[10], which was used to inform frailty-status transitions and BMI estimates. However, the BMI distribution in SLAS-2 included few individuals in the obese II category, limiting its ability to assess the impact of obesity on frailty transitions directly. Therefore, we first estimated transition probabilities considering risk factors including age, gender, and ethnicity, and then incorporated odds ratios of normal weight and other BMI categories on pre-frailty and frail status from other studies[10,11] conducted in Singapore to estimate transition probabilities across BMI categories. This approach allowed us to better reflect the effect of excess BMI, including extreme obesity, which has been shown to be associated with increased frailty risk in studies[12–16].

BMI Intervention Modelling

The means and standard deviations of BMI among robust, pre-frail, and frail older individuals from the follow-up subsample of 1162 community-dwelling older Singaporeans from SLAS-2 were presented as 24.5 ± 3.5, 23.6 ± 4.3, 22.9 ± 4.5 (mean $\pm$ standard deviation), respectively[10]. Based on the proportions of robust (52.6%), pre-frail (44.2%), and frail (3.2%) individuals in the SLAS-2 sample[10], we generated three datasets comprising 5,210 robust, 4,460 pre-frail, and 329 frail individuals, respectively. For each dataset, as individual-level SLAS-2 data were not available, we simulated BMI values using truncated normal distributions, ensuring that the means and standard deviations were equal to 24.5 and 3.5 for robust individuals, 23.6 and 4.3 for pre-frail individuals, and 22.9 and 4.5 for frail individuals, respectively. We combined the three datasets and analysed BMI values and frailty states using logistic regression models to estimate the association of BMI categories including underweight (<18·5 kg/m^2), normal weight (18·5–22·9 kg/m^2), overweight (23·0–24·9 kg/m^2), and obesity I (25·0–29·9 kg/m^2) on being pre-frail and frail. Since the proportion of simulated samples with BMI in the Obese II category (≥30.0 kg/m^2) was too low to adequately assess its effect on frailty states, we used the observed proportions of robust, pre-frail, and frail individuals within the Obese II category in the sample of 2102 Singapore residents from the Well-being of the Singapore Elderly study (WBSE)[11] to estimate its effect on pre-frailty and frailty compared to normal weight and to calculate the corresponding odds ratios.

To explore the association of BMI categories on pre-frailty and frailty, we fitted two separate logistic regression models using the BMI category as the predictor and frailty status as the binary outcome. The models followed the logistic regression form:

$$logit\big(P(Y=1)\big) = \beta_0 + \beta_1 Underweight + \beta_2 Overweight + \beta_3 Obesity\ I,$$

where $Y = 1$ represents the outcome of interest, and BMI was treated as a categorical predictor with four levels: underweight (<18·5 kg/m^2), normal weight (18·5–22·9 kg/m^2; reference category), overweight (23·0–24·9 kg/m^2), and obesity I (25·0–29·9 kg/m^2).

In the first model, we examined the association between BMI and the likelihood of being pre-frail versus robust. We included only robust and pre-frail individuals in the dataset, coding the outcome variable as 0 for robust and 1 for pre-frail.

In the second model, we assessed the association between BMI and the likelihood of being frail versus pre-frail. This model included only pre-frail and frail individuals, with the outcome coded as 0 for pre-frail and 1 for frail.

To incorporate BMI into the frailty transition model, we estimated transition rates for each BMI category by using odds ratios as BMI-specific relative transition-rate multipliers applied to the transition rate of the reference category, based on the studies demonstrating that odds ratios are reliable estimates of incidence rate ratios[17–19]. Then we converted these transition rates into annual transition probabilities for each BMI category within the continuous-time Markov framework[20]. This approach allowed us to incorporate BMI into the estimation of transition probabilities in a mathematically justified and more comprehensive manner than relying solely on individual-level data from a sample with limited BMI variation. We additionally found that utilising either ratio led to very modest differences in outputs.

Calibration and Validation Checks

We used grid search to evaluate different allocations of robust, pre-frail, and frail individuals for each age, gender, and ethnicity in 2011, and their transitions between 2011 and 2016 in DEMOS. These generated multiple datasets for the Bayesian multistate model to estimate transition probabilities. We then applied each set of transition probabilities in DEMOS to project individuals' frailty states from 2011 to 2016. As an internal calibration check, we compared the modelled prevalences of pre-frailty and frailty among individuals aged 60 years or over with the observed prevalences from the SLAS-2 follow-up cohort[10], and selected the set of transition probabilities that minimised the total error. In this way, our estimated prevalence of pre-frailty and frailty (48.9% and 7.5%) matched the observed prevalences.

Estimation of Healthcare Utilisation

Table S2: Means of Healthcare Utilisation. The six-month means before and after frailty-status assessment were collected from the Population Health Index study covering two periods: six months before and six months after the observation of frailty statuses[21].

Healthcare Utilisation by Setting	Frailty State	six-month mean before assessment	six-month mean after assessment
Specialist Outpatient Clinic Visits (SOCV)	Robust	1·22	1·21
	Pre-frail	2·40	2·03

	Frail	3·92	5·08
Emergency Department Visits (EDV)	Robust	0·09	0·05
	Pre-frail	0·18	0·19
	Frail	0·57	0·47
Hospitalisations	Robust	0·04	0·03
	Pre-frail	0·14	0·12
	Frail	0·51	0·53

Projection Results across Weight Management Scenarios

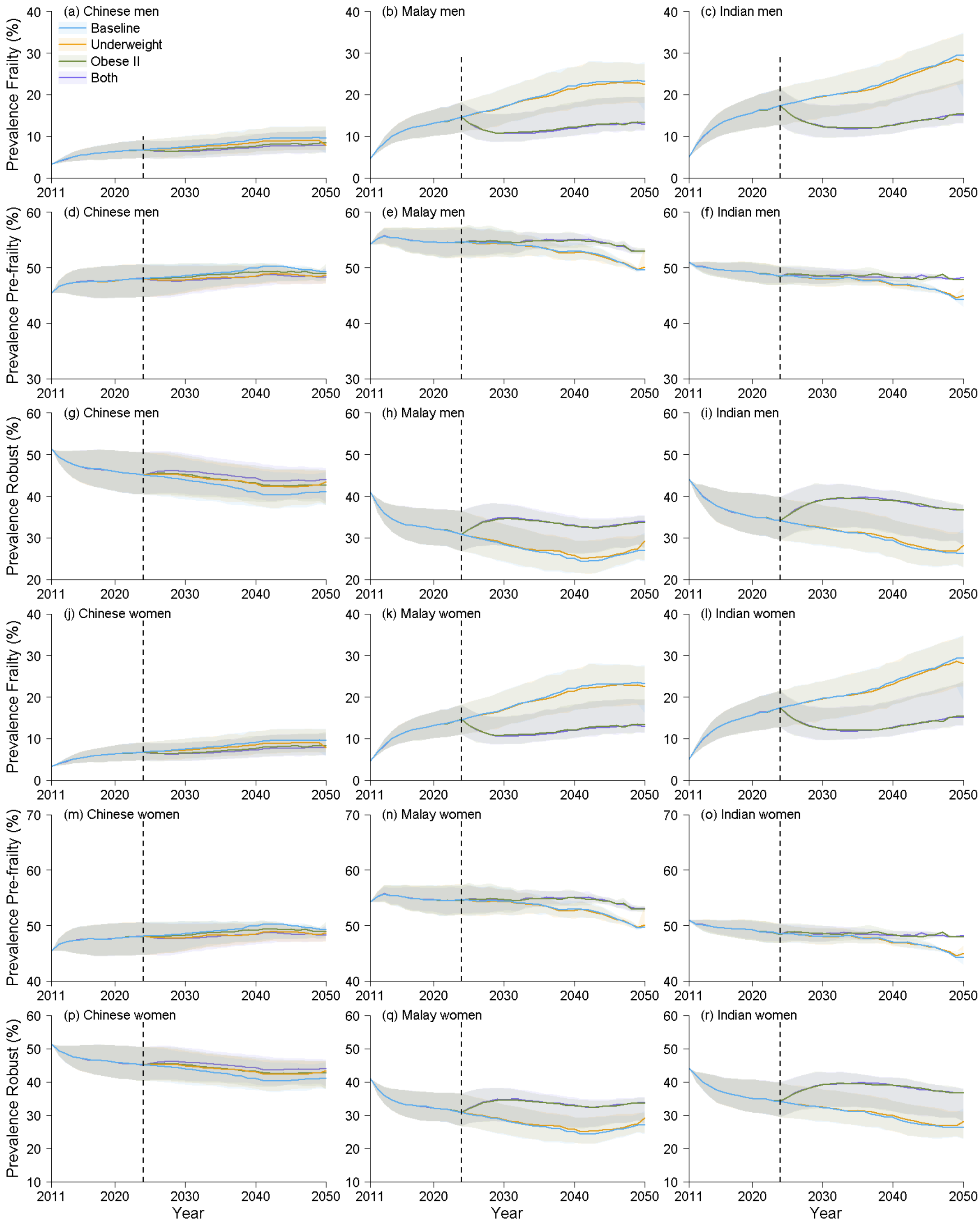

Figure S5. Projected prevalences by frailty status under weight management scenarios, stratified by ethnicity and gender. Posterior means and 95% credible intervals are shown as shaded areas from 2011 to 2050 for Chinese, Malay, and Indian men and women under four scenarios: baseline, underweight to normal weight intervention ("Underweight"), obese II to obese I intervention ("Obese II"), and the combination of both Underweight and Obese II strategies ("Both"). Panels show: (a) frail prevalence for Chinese men, (b) frail prevalence for Malay men, (c) frail prevalence for Indian men, (d) pre-frail prevalence for Chinese men, (e) pre-frail prevalence for Malay men, (f) pre-frail prevalence for Indian men, (g) robust prevalence for Chinese men, (h)

robust prevalence for Malay men, (i) robust prevalence for Indian men, (j) frail prevalence for Chinese women, (k) frail prevalence for Malay women, (l) frail prevalence for Indian women, (m) pre-frail prevalence for Chinese women, (n) pre-frail prevalence for Malay women, (o) pre-frail prevalence for Indian women, (p) robust prevalence for Chinese women, (q) robust prevalence for Malay women, (r) robust prevalence for Indian women. Uncertainty intervals were derived from n=360 stochastic projections per scenario.

References

1. Department of Statistics Singapore. SingStat Table Builder [Internet]. 2025 [cited 2025 Feb 17]. Singapore Residents By Single Year Of Age, Ethnic Group And Sex, At End June. Available from: https://tablebuilder.singstat.gov.sg/table/TS/M810801

2. Department of Statistics Singapore. SingStat [Internet]. 2025 [cited 2025 May 4]. Births and Fertility - Latest Data. Available from: http://www.singstat.gov.sg/find-data/search-by-theme/population/births-and-fertility/latest-data

3. Department of Statistics Singapore. SingStat [Internet]. 2025 [cited 2025 May 4]. Death and Life Expectancy - Latest Data. Available from: http://www.singstat.gov.sg/find-data/search-by-theme/population/death-and-life-expectancy/latest-data

4. Song H, Jin S, Yang GJ, Lim NWH, Lim JT, Dickens BL. Modelling the impact and cost-effectiveness of upcoming Zika virus vaccines on congenital Zika syndrome. Vaccine. 2025 Feb 27;48:126728. doi:10.1016/j.vaccine.2025.126728

5. Jackson C, Zapata-Diomedi B, Woodcock J. Bayesian multistate modelling of incomplete chronic disease burden data. J R Stat Soc Ser A Stat Soc. 2023 Jan;186(1):1–19. doi:10.1093/jrsssa/qnac015 PubMed PMID: 36883132; PubMed Central PMCID: PMC7614284.

6. Jackson C. disbayes: Bayesian Multi-State Modelling of Chronic Disease Burden Data [Internet]. 2022 [cited 2026 May 12]. Available from: https://CRAN.R-project.org/package=disbayes. doi:10.32614/CRAN.package.disbayes

7. Wood SN. Thin Plate Regression Splines. Journal of the Royal Statistical Society Series B: Statistical Methodology. 2003 Feb 1;65(1):95–114. doi:10.1111/1467-9868.00374

8. Wood SN. Generalized Additive Models: An Introduction with R. 2nd ed. New York: Chapman and Hall/CRC; 2017. 496 p. doi:10.1201/9781315370279

9. Merchant RA, Chen MZ, Tan LWL, Lim MY, Ho HK, van Dam RM. Singapore Healthy Older People Everyday (HOPE) Study: Prevalence of Frailty and Associated Factors in Older Adults. Journal of the American Medical Directors Association. 2017 Aug 1;18(8):8. doi:10.1016/j.jamda.2017.04.020

10. Wei K, Thein FS, Nyunt MSZ, Gao Q, Wee SL, Ng TP. Nutritional and Frailty State Transitions in the Singapore Longitudinal Aging Study. J Nutr Health Aging. 2018;22(10):10. doi:10.1007/s12603-018-1096-3 PubMed PMID: 30498830.

11. Vaingankar JA, Chong SA, Abdin E, Picco L, Chua BY, Shafie S, et al. Prevalence of frailty and its association with sociodemographic and clinical characteristics, and resource utilization in a population of Singaporean older adults. Geriatr Gerontol Int. 2017 Oct;17(10):10. doi:10.1111/ggi.12891 PubMed PMID: 27576598.

12. Barzilay JI, Cotsonis GA, Walston J, Schwartz AV, Satterfield S, Miljkovic I, et al. Insulin Resistance Is Associated With Decreased Quadriceps Muscle Strength in Nondiabetic Adults Aged ≥70 Years. Diabetes Care. 2009 Apr 1;32(4):736–8. doi:10.2337/dc08-1781

13. Blaum CS, Xue QL, Michelon E, Semba RD, Fried LP. The association between obesity and the frailty syndrome in older women: the Women’s Health and Aging Studies. J Am Geriatr Soc. 2005 Jun;53(6):927–34. doi:10.1111/j.1532-5415.2005.53300.x PubMed PMID: 15935013.

14. Woods NF, LaCroix AZ, Gray SL, Aragaki A, Cochrane BB, Brunner RL, et al. Frailty: emergence and consequences in women aged 65 and older in the Women's Health Initiative Observational Study. J Am Geriatr Soc. 2005 Aug;53(8):1321–30. doi:10.1111/j.1532-5415.2005.53405.x PubMed PMID: 16078957.

15. Stenholm S, Strandberg TE, Pitkälä K, Sainio P, Heliövaara M, Koskinen S. Midlife Obesity and Risk of Frailty in Old Age During a 22-Year Follow-up in Men and Women: The Mini-Finland Follow-up Survey. J Gerontol A Biol Sci Med Sci. 2014 Jan 1;69(1):73–8. doi:10.1093/gerona/glt052

16. Hubbard RE, Lang IA, Llewellyn DJ, Rockwood K. Frailty, body mass index, and abdominal obesity in older people. J Gerontol A Biol Sci Med Sci. 2010 Apr;65(4):377–81. doi:10.1093/gerona/glp186 PubMed PMID: 19942592.

17. Miettinen O. Estimability and estimation in case-referent studies. Am J Epidemiol. 1976 Feb;103(2):226–35. doi:10.1093/oxfordjournals.aje.a112220 PubMed PMID: 1251836.

18. Greenland S, Thomas DC. On the need for the rare disease assumption in case-control studies. Am J Epidemiol. 1982 Sep;116(3):547–53. doi:10.1093/oxfordjournals.aje.a113439 PubMed PMID: 7124721.

19. Newman SC. Odds ratio estimation in a steady-state population. Journal of Clinical Epidemiology. 1988 Jan 1;41(1):59–65. doi:10.1016/0895-4356(88)90009-1

20. Hanley JA. Going from the incidence rate (or hazard rate) function to the risk function [Internet]. 2018. Available from: https://jhanley.biostat.mcgill.ca/bios601/SurvivalAnalysis/IncidenceFunctionToRisk2018.pdf

21. Ge L, Yap CW, Heng BH, Tan WS. Frailty and healthcare utilisation across care settings among community-dwelling older adults in Singapore. BMC Geriatrics. 2020 Oct 6;20(1):1. doi:10.1186/s12877-020-01800-8
