# Supplementary material for: Bayesian multistate modelling of prefrailty and frailty using microsimulation with weight management interventions for prevention in Asian settings
Source: Commun Med (Lond). 2026 Jul 4;6:560. doi: 10.1038/s43856-026-01756-7 (PMC13713980; doi:10.1038/s43856-026-01756-7)
Supplement: Supplementary file 2 [file 43856_2026_1756_MOESM2_ESM.docx]

**Description of Additional Supplementary Files**

File name: Supplementary Data 1-5

Description: Source data
